# Association test using Copy Number Profile Curves (CONCUR) enhances power in rare copy number variant analysis

**DOI:** 10.1371/journal.pcbi.1007797

**Published:** 2020-05-04

**Authors:** Amanda Brucker, Wenbin Lu, Rachel Marceau West, Qi-You Yu, Chuhsing Kate Hsiao, Tzu-Hung Hsiao, Ching-Heng Lin, Patrik K. E. Magnusson, Patrick F. Sullivan, Jin P. Szatkiewicz, Tzu-Pin Lu, Jung-Ying Tzeng

**Affiliations:** 1 Department of Statistics, North Carolina State University, Raleigh, North Carolina, United States of America; 2 Institute of Epidemiology and Preventive Medicine, National Taiwan University, Taipei, Taiwan; 3 Department of Medical Research, Taichung Veterans General Hospital, Taiwan; 4 Department of Medical Epidemiology and Biostatistics, Karolinska Institutet SE-171 77 Stockholm, Sweden; 5 Department of Genetics, University of North Carolina at Chapel Hill, Chapel Hill, North Carolina, United States of America; 6 Bioinformatics Research Center, North Carolina State University, Raleigh, North Carolina, United States of America; 7 Department of Statistics, National Cheng-Kung University, Tainan, Taiwan; Carnegie Mellon University, UNITED STATES

## Abstract

Copy number variants (CNVs) are the gain or loss of DNA segments in the genome that can vary in dosage and length. CNVs comprise a large proportion of variation in human genomes and impact health conditions. To detect rare CNV associations, kernel-based methods have been shown to be a powerful tool due to their flexibility in modeling the aggregate CNV effects, their ability to capture effects from different CNV features, and their accommodation of effect heterogeneity. To perform a kernel association test, a CNV locus needs to be defined so that locus-specific effects can be retained during aggregation. However, CNV loci are arbitrarily defined and different locus definitions can lead to different performance depending on the underlying effect patterns. In this work, we develop a new kernel-based test called CONCUR (i.e., copy number profile curve-based association test) that is free from a definition of locus and evaluates CNV-phenotype associations by comparing individuals’ copy number profiles across the genomic regions. CONCUR is built on the proposed concepts of “copy number profile curves” to describe the CNV profile of an individual, and the “common area under the curve (cAUC) kernel” to model the multi-feature CNV effects. The proposed method captures the effects of CNV dosage and length, accounts for the numerical nature of copy numbers, and accommodates between- and within-locus etiological heterogeneity without the need to define artificial CNV loci as required in current kernel methods. In a variety of simulation settings, CONCUR shows comparable or improved power over existing approaches. Real data analyses suggest that CONCUR is well powered to detect CNV effects in the Swedish Schizophrenia Study and the Taiwan Biobank.

## Author summary

Copy number variants comprise a large proportion of variation in human genomes. Large rare CNVs, especially those disrupting genes or changing the dosages of genes, can carry relatively strong risks for neurodevelopmental and neuropsychiatric disorders. Kernel-based association methods have been developed for the analysis of rare CNVs and shown to be a valuable tool. Kernel methods model the collective effect of rare CNVs using flexible kernel functions that capture the characteristics of CNVs and measure CNV similarity of individual pairs. Typically kernels are created by summarizing similarity within an artificially defined “CNV locus” and then collapsing across all loci. In this work, we propose a new kernel-based test, CONCUR, that is based on the CNV location information contained in standard processing of the variants and which obviates the need for arbitrarily defined CNV loci. CONCUR quantifies similarity between individual pairs as the common area under their copy number profile curves and is designed to detect CNV dosage, length and dosage-length interaction effects. In simulation studies and real data analysis, we demonstrate the ability of the CONCUR test to detect CNV effects under diverse CNV architectures with power and robustness over existing methods.

This is a *PLOS Computational Biology* Methods paper.

## Introduction

Copy number variants (CNVs) are unbalanced structural variants that are typically 1 kilobase pair (kb) in size or larger and are comprised of more or fewer copies of a region of DNA with respect to the reference genome. CNVs are typically characterized by two descriptive features. The first feature is CNV dosage, or the total number of copies present, with > 2 copies corresponding to duplications and < 2 copies corresponding to deletions. The second is the CNV length, typically measured in base pairs (bp) or kilobase pairs.

CNVs are important risk factors for many human diseases and traits, including Crohn’s disease, HIV susceptibility, and body mass index [1–3]. Large and rare CNVs are particularly implicated in neuropsychiatric disorders including autism spectrum disorder, schizophrenia, bipolar disorder, and attention deficit disorder [4]. For example, multiple studies have confirmed a greater burden of rare CNVs in schizophrenia cases compared with normal controls, both genome-wide and in specific neurobiological pathways important to schizophrenia (e.g., calcium channel signaling and binding partners of the fragile X mental retardation protein).

Rare CNVs (e.g., < 1% frequency) in the genome are intractable to test individually for disease association and instead are examined with collapsing methods. Collapsing methods summarize variant characteristics across multiple variants in a targeted region, such as a gene set, a chromosome or the whole genome, and perform a test of the collective CNV effects on the phenotype. By accumulating information across multiple rare variants, collapsing methods can have enhanced power to detect the effects of rare CNVs that are difficult to detect individually but collectively have a significant impact. Collapsing tests for rare CNVs are primarily built on the foundation of rare single nucleotide polymorphism (SNP) association tests but with additional complexity to accommodate the length and dosage features of CNVs. As with SNPs, the effects of CNVs can vary between loci, and collapsing methods demonstrate improved power as they account for this heterogeneity. However, CNV collapsing tests also need to account for within-locus heterogeneity due to differential dosage effects or length effects within a CNV region.

Similar to SNP collapsing tests, there are two families of tests for rare CNV analysis: burden-based methods and kernel-based methods. Burden-based tests, e.g., Raychaudhuri et al. [5], summarize the CNV features of an individual via total CNV counts or average length and model the CNV effects as fixed effects, assuming etiological homogeneity of features across multiple CNVs in a targeted region. Kernel-based tests, such as CCRET [6] and CKAT [7], aggregate CNV information via genetic similarity based on certain CNV features and model CNV effects as random effects to account for the between-locus etiological heterogeneity. By design, burden tests are optimal when the association signal is driven by homogeneous effects across CNVs, and kernel-based tests are optimal in the presence of etiological heterogeneity. Burden testing often involves multiple analyses on subsets of CNVs according to their dosage (e.g., deletions only or duplications only) or size (e.g. > 100kb, > 500kb) to increase homogeneity; whereas, kernel-based tests do not have such requirements.

In this work, we focus on kernel-based methods, as etiological heterogeneity is becoming a more practically encountered scenario due to high-resolution CNV detection technologies permitting the detection of CNVs of smaller length. In kernel-based association tests, the association between CNVs and the trait is evaluated by examining the correlation between trait similarity and CNV similarity quantified in a kernel. For kernel construction, we can refer to kernel-based tests for SNPs. Since SNPs are evaluated at the same single base-pair position (referred to as a locus) across individuals, it is natural to assess similarity locus-by-locus and aggregate the locus-level similarity over all loci in the target region to obtain an overall measure of SNP similarity. A locus unit for CNVs, however, is not so obvious since CNVs span multiple base pairs and may overlap partially between individuals.

To address this issue, standard CNV kernel-based tests construct CNV regions (CNVRs). For example, the CNV Collapsing Random Effects Test (CCRET) [6], developed previously by our group, defines CNVRs by a user-specified amount of CNV overlapping among different individuals (e.g., Fig 1 of Tzeng et al. [6]). CNV similarity between an individual pair is quantified first within each CNVR, and then CNVR-level similarity is summed over all CNVRs in the target region to characterize overall CNV similarity. However, a drawback of this approach is that CNVRs defined in this fashion are contingent on the unique CNV overlapping patterns among individuals in a study, and the defined CNVRs can vary from one study to another. The arbitrary choice of overlapping threshold also impacts the formation of locus units and consequently how the “between-locus” and “within-locus” heterogeneous effects of CNVs are accounted for.

To avoid the issues introduced by arbitrarily defined CNVRs as in CCRET, the CNV Kernel Association Test (CKAT) [7] adopts a different strategy to quantify CNV similarity between two individuals. Specifically, CKAT allows users to define the CNVR as a biologically relevant region, e.g., a chromosome. CKAT also introduces a new kernel function to measure CNV similarity based on both dosage and length features between two CNV events. This CNV-level similarity is then aggregated to derive a measure of CNVR-level similarity using a shift-by-one scanning algorithm that “aligns” CNVs in two profiles based on their ordinal position. A multiple-testing correction is applied if multiple CNVRs are involved in the targeted region. Although the new strategy bypasses the need for an arbitrarily defined locus unit, the scanning alignment may yield unreliable results if CNVRs are too large. Additionally, CKAT aligns pairs of CNVs based on their ordinal position, which may or may not optimally capture similarity dependent on the CNV signal sources. There may also be computational considerations with a scanning algorithm when *n* is large.

**Fig 1:**
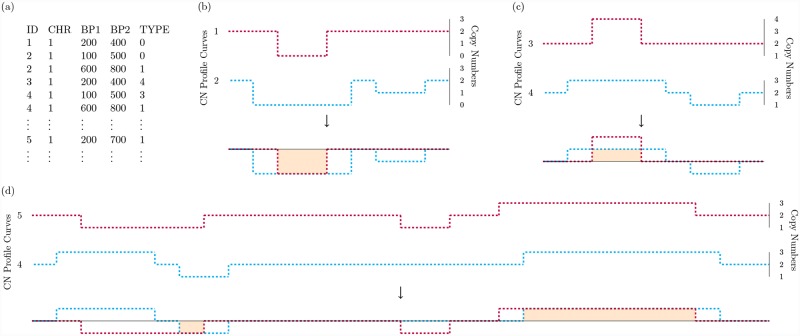
Diagram of copy number profile curves and common area under the curve. (a) Example of CNV data in standard PLINK format describing profiles of individuals in a small region of chromosome 1. (b) Copy number (CN) profile curves illustrating the cAUC between individuals with overlapping deletions of dosage 0. (c) CN profile curves illustrating the cAUC between individuals with overlapping duplications of dosage 3 and 4. (d) CN profile curves which show the cAUC between two individuals who have overlapping deletions of dosage 1 and overlapping duplications of dosage 3, so that the cAUC between the individuals is the sum of the two areas.

To address these challenges in quantifying CNV similarity using kernel-based methods, in this work we propose a new approach called the Copy Number profile Curve-based (CONCUR) association test. Based on the concept of copy number (CN) profile curves (Fig 1), the CONCUR association test naturally incorporates both CNV dosage and length features and can capture their main effects as well as dosage-length interactions. Additionally, building the kernel based on CN profile curves permits the quantification of CNV similarity without the need of pre-specified locus units. Moreover, CNV length may be incorporated flexibly in units which are supported in good resolution by the sequencing technology or which are computationally stable. Like CCRET and CKAT, the test is built in the framework of kernel machine regression and is powerful under heterogeneous signals and can adjust for confounders. We use simulation studies to demonstrate the improved power CONCUR over existing kernel-based methods in a variety of settings and illustrate the practical utility of CONCUR by conducting pathway analysis on data from the Swedish Schizophrenia Study and the Taiwan Biobank.

## Results

### Overview of CONCUR

CONCUR assesses the collective effects of rare CNVs on a phenotype in a kernel machine regression framework where the kernel construction does not require a pre-defined CNV locus or region. As such, CONCUR is built on two major components: (a) the CN profile curve, with which we describe an individual’s CNVs across the genome or a region of interest; and (b) the common area under the curve (cAUC) kernel, with which we measure CNV similarity between individuals and characterize the CNV effects on the phenotype. In a CN profile curve (e.g., Fig 1), CNV dosage is shown on the y-axis as jumps or troughs diverging from a baseline of 2 copies. The start and end points of the jumps and troughs correspond to the start and end locations of the CNV and are shown on the x-axis. At genomic locations where there are no CNV events, the y-axis (dosage) takes value 2 (i.e., the baseline value). CN profile curves are intended to be a visualization of CNV activity and concurrence across samples and contribute to the CONCUR method through the concept of cAUC.

By superimposing two CN profile curves, we identify regions of overlapping CNVs of the same type (i.e., deletion or duplication) and propose to use the common area under the curve (cAUC) to quantify CNV similarity between two individuals. To implement the idea, first the raw dosage values in the CN profile curve are centered and scaled to obtain the duplication profile curve and deletion profile curve. Let *d* denote the dosage of a CNV. The scaling and centering can be achieved by the dosage transform functions: *a*^*Dup*^(*d*) = |2 − *d*|^*c*^ for duplications and 0 otherwise, and *a*^*Del*^(*d*) = |2 − *d*|^*c*^ for deletions and 0 otherwise, where *c* is some pre-specified constant. Second, we superimpose the duplication profile curves of two individuals and note the overlapping regions where both curves are non-zero. Third, for each overlapping region, we multiply the minimum of the two respective transformed dosage values by the length of the overlap, and save this measure of “area of commonality”. Finally, we calculate the cAUC between two individuals as the sum of all such areas of commonality in their duplication profile curves plus the sum of all areas in their deletion profile curves. Fig 1 and S1 Fig. illustrate the cAUCs between various pairs of individuals when setting *c* = 1 in the dosage transform functions, *a*^*Dup*^(*d*) and *a*^*Del*^(*d*). Generally speaking, the cAUC between individuals with overlapping CNVs of the same dosage *d* is the overlapping length times |*d* − 2|. For example, for individuals with overlapping CNVs of dosage 0 (Fig 1(b)), the cAUC is the overlapping length times |0 − 2|. The cAUC between individuals with overlapping CNVs of the same type but different dosages, *d*_1_ and *d*_2_, is the length of the overlap times |*d*_1_ − *d*_2_| (e.g., dosages of 3 versus 4 in Fig 1(c)). If there are multiple overlaps in the individuals’ CN profile curves, the cAUC between two individuals is the sum of all areas of commonality (e.g., sum of shaded regions in Fig 1(d)). The cAUC kernel measures similarity in both CNV length and dosage and hence characterizes the joint dosage and length effects. Using the semi-parametric kernel machine regression framework, CONCUR regresses the trait values on CNV effects captured by the cAUC kernel, and evaluates the association between traits and CNV profiles via a score-based variance component test.

### Simulation studies

We conducted three sets of simulations: whole genome analysis based on **T**win**G**ene **p**seudo-CNV (TGP) data (referred to as TGP-WG simulations); chromosome 1 analysis based on TGP data (referred to as TGP-Chr1 simulations); and chromosome 1 analysis based on **T**ai**w**an **B**iobank (TWB) CNV data (referred to as TWB-Chr1 simulations). The dosage values are integers in the TGP dataset and are continuous in TWB dataset. With these simulations, we evaluated the performance of CONCUR, CCRET, and CKAT under various signal patterns and different sources of effect heterogeneity. For reference, a table of all simulation settings is provided in S1 Table.

To implement CCRET, we applied the functions from the CCRET package to convert the PLINK data to CCRET design matrices and computed the dosage kernel matrix. We used 1-bp overlapping of CNVs among different individuals to form CNVRs as in the CCRET paper [6]; that is, as long as CNVs of different individuals overlapped by ≥1bp, it was considered an “overlap”. For CKAT, we designated a chromosome as a CNVR and performed an association test for each CNVR using the CKAT package. CNV lengths within each chromosome were scaled to be in [0, 1] by dividing by the range of CNV activity in each chromosome, i.e., the maximal ending position minus the minimal starting position of observed CNVs on each chromosome. The Gaussian kernel scaling parameter was set to be 1. In the TGP-WG simulations, as there were 22 CKAT p-values corresponding to the 22 chromosomes, we took the minimum p-value and used Bonferroni’s procedure to compute the adjusted p-value for multiple testing. Finally, we built the CCRET and CKAT kernels using CNVs’ categorical dosage (duplication, deletion, and normal) as required in the software packages. To assure comparability, CONCUR was built using categorical dosages (referred to as CONCUR_cat). We also implemented CONCUR using the original integer dosage values (referred to as CONCUR_int) in the TGP-Chr1 simulations, and using the original continuous dosages (referred to as CONCUR_cont) in the TWB-Chr1 simulation.

#### Datasets used for simulations

The TwinGene pseudo (TGP) CNV dataset of 2000 individuals is publicly available at https://www4.stat.ncsu.edu/~jytzeng/Software/CCRET/software_ccret.php. Autosome-wide pseudo-CNV data were simulated by mimicking the CNV profiles of unrelated individuals in the TwinGene study [8], and the details are described in Tzeng et al. [6]. Briefly, the TwinGene study used a cross-sectional sampling design and included over 6,000 unrelated subjects born between 1911 and 1958 from the Swedish Twin Registry [9, 10]. CNV calls were generated using the Illumina OmniExpress BeadChip for 72,881 SNP markers and using PennCNV (version June 2011) [11] as the CNV calling algorithm with recommended model parameters. From the full callset, high quality rare CNVs (frequency < 1% and size > 100kb) were extracted to form the simulation pool for the pseudo-CNV data. CNV dosages in this dataset are integers (0, 1, 2, 3, and 4). In the TGP whole genome data (chromosome 1 to chromosome 22), each of the 2000 individuals has at least one CNV, and in the chromosome 1 analysis, 291 individuals have CNV activity.

The Taiwan Biobank (TWB) CNV dataset is from the Taiwan Biobank project https://www.twbiobank.org.tw/new_web/. This data was studied in the real data analysis and further details regarding it are shared in the section **CNV analysis on triglycerides in the Taiwan Biobank**. CNV dosage values in this dataset are continuous with dosages ≥ 2.3 indicating duplications and ≤ 1.7 indicating deletions. Out of the 11,664 individuals who were included in the real data analysis, we took a random sample of 2000 individuals and kept their data for all CNVs in chromosome 1. In this sample, 1432 individuals out of the 2000 had CNV activity in chromosome 1.

#### Simulation design

For the purpose of simulating phenotypes, we constructed “CNV segments” based on the CNVs in the focal dataset. The endpoints of the segments correspond to locations where a CNV in any one of the samples begins or ends, resulting in segments that contain either one or more intersecting CNVs. Within a segment, CNV dosage of an individual is a constant, and CNVs across individuals may have different dosages though they share the same starting and ending positions defined by the boundaries of the segment. Note that different segments will naturally have different lengths. In the simulation studies, we built design matrices **Z**^*Dup*^, **Z**^*Del*^, and **Z**^*Len*^ which codified CNV features by segment in the CNV dataset. The dosage matrices, **Z**^*Dup*^ and **Z**^*Del*^, took value 0 for those individuals without CNVs in the segment and were coded as the number of additional or missing copies comprising the CNV otherwise. **Z**^*Len*^ was the length of the CNV segment in kb for individuals with CNV events and was 0 for individuals without CNVs in the segment.

A case-control phenotype was generated from the logistic model

$$\begin{array}{ccc} \text{logit}(\mathrm{Pr}\left(Y_{i}=1\right)) & = & \gamma_{0}+\beta_{X}X_{i}+\sum_{j=1}^{R}\beta_{j}^{Dup}Z_{ij}^{Dup}+\sum_{j=1}^{R}\beta_{j}^{Del}Z_{ij}^{Del}+\sum_{j=1}^{R}\beta_{j}^{Len}Z_{ij}^{Len}\\ & + & \sum_{j=1}^{R}\beta_{j}^{Dup*Len}Z_{ij}^{Dup}Z_{ij}^{Len}+\sum_{j=1}^{R}\beta_{j}^{Del*Len}Z_{ij}^{Del}Z_{ij}^{Len}, \end{array}$$ (1)

where *Z*_*ij*_^•^ is the (i,j) entry of matrix **Z**^•^, *i* = 1, ⋯, *N* indexes individuals, and *j* = 1, ⋯, *R* indexes CNV segments. A binary covariate *X*_*i*_ was simulated from Bernoulli(0.5) for each individual. $\beta_{j}^{Dup}$ and $\beta_{j}^{Del}$ are the log odds ratios of segment *j* for the presence of a CNV versus the absence. Likewise, $\beta_{j}^{Len}$ controls the effect of CNV length in segment *j*, and $\beta_{j}^{Dup*Len}$ and $\beta_{j}^{Del*Len}$ allow the effects of CNV length to differ by dosage. *β*_*j*_^•^ (or < 0) corresponds to a deleterious (or protective) CNV effect, and *β*_*j*_^•^ was set to 0 in non-causal segments. We set *β_X_* = log(1.1) and *γ*_0_ = -2, which corresponds to a baseline disease rate of roughly 0.12. We also fixed $\beta_{j}^{Len}=0$ to reflect the observation that length tends to act like an effect modifier of dosage effects.

In the TGP-WG simulations, we generated phenotypes from CNV dosage × length effects and from dosage-only effects. We chose these signals to roughly replicate the simulation settings applied to assess CKAT in [7] (dosage × length signal) and CCRET in [6] (dosage signal). In the TGP-Chr1 and TWB-Chr1 simulations, signals were generated from CNV dosage × length effects. Below we describe the settings for the Chr1-based simulations; the TGP-WG simulations basic settings are similar and are detailed in S2 Appendix.

In the Chr1-based simulations, we considered three types of causal effects: causal effects from both duplications and deletions, causal effects from duplications only, and causal effects from deletions only. Under each effect type, we designated varying percentages of the causal segments to be deleterious (D) or protective (P). When both duplications and deletions were causal, the settings included (D_Dup_, P_Dup_, D_Del_, P_Del_) = (90, 10, 90, 10), i.e., both causal duplications and causal deletions had 90% deleterious and 10% protective effects, as well as (90, 10, 10, 90) and (10, 90, 90, 10). In the scenarios where duplications or deletions alone were causal, settings included (D_•_, P_•_) = (90,10), (50,50), and (10,90).

In the TGP-Chr1 simulations, we randomly selected 40 segments across chromosome 1 to be causal, comprised of 20 segments containing ≥1 duplication and 20 segments containing ≥1 deletion. As the segments were formed purely based on the relative CNV patterns among individuals and could be very short in length, we also required causal segments to be at least as long as the median length of all segments of that type (35kb for duplications, 46kb for deletions) to ensure that they had realistic lengths. We allowed for the possibility of duplication and deletion effects arising from the same location, and used categorical dosages to simulate the length-dosage effects. That is, when simulating phenotypes using Eq 1, for individual *i* and segment *j*, we set $Z_{ij}^{Dup}=1$ if a duplication was present in the segment and 0 otherwise, and set $Z_{ij}^{Del}=1$ if a deletion was present in the segment and 0 otherwise. We implemented CONCUR in all TGP-Chr1 simulations using the CONCUR_cat kernel as well as the CONCUR_int kernel. We refer to the three scenarios described here (i.e., effects from duplications only, from deletions only, and from combined effects) as TGP-Chr1(a) to distinguish it from the sensitivity analyses introduced below.

In the scenario with causal effects from duplications and deletions combined, we considered two additional scenarios as sensitivity analyses: TGP-Chr1(b) examines the methods’ performance under inaccuracy in the called end-points of CNVs; and TGP-Chr1(c) imposes a more rare baseline disease rate. In TGP-Chr1(b), we added random uniformly distributed errors to the endpoints (BP1 and BP2) of all CNVs. The CNV endpoints in the error-added data differed from the CNV endpoints in the true data used to generate phenotypes by up to ±2.5% of the total length of the CNV. In TGP-Chr1(c), we set *γ*_0_ = −3 to lower the baseline disease rate to roughly 0.05.

In the TWB-Chr1 simulations, we randomly selected 600 CNV segments across chromosome 1 to be causal, comprised of 300 segments containing ≥1 duplication and 300 segments containing ≥1 deletion. We imposed similar criteria on the length of causal segments as in the TGP-Chr1 simulations, and we allowed for duplication and deletion effects from the same location. Unlike the TGP-Chr1 simulations, here we used continuous dosages to simulate the length-dosage effects in all three scenarios (referred to as TWB-Chr1(a) simulations). In the scenario with combined duplication and deletion causal effects, we also generated signals from categorical dosages (referred to as TWB-Chr1(b) simulations). When simulating phenotypes based on continuous dosage signals using Eq 1, we constructed the dosage matrices such that for a CNV of dosage *d* in segment *j* for individual *i*, $Z_{ij}^{Dup}=\left|d-2\right|$ if a duplication was present and 0 otherwise, and $Z_{ij}^{Del}=\left|d-2\right|$ if a deletion was present and 0 otherwise. We applied the CONCUR_cont kernel and CONCUR_cat kernel in both settings (a) and (b) of the TWB-Chr1 simulations to evaluate their robustness to signals arising from dosage values of the same type (categorical or continuous) or of an incongruent type.

We implemented case-control sampling to obtain 2000 cases and 2000 controls for each simulation replication. Type I error rates were evaluated based on 5000 replications, and power was estimated based on 300 replications at each effect size. For all methods, we adjusted for a simulated binary covariate as a fixed effect in the kernel machine regression. We employed the small-sample variance components test of Chen et al. [12] and obtained p-values using Davies’ method [13] as implemented in the CKAT R functions.

#### Results of TGP-WG simulations

The type I error rates of CONCUR, CCRET, and CKAT were examined at nominal levels of 0.01, 0.05, and 0.1 in the TGP-WG simulations (Table 1). All methods had type I error rates around the nominal level.

**Table 1:** Type I error rates. Type I error rates of three CNV tests evaluated based on 5000 replications.

Nominal level	*CONCUR*	*CCRET*	*CKAT*
0.01	0.010	0.008	0.009
0.05	0.045	0.047	0.049
0.10	0.096	0.093	0.092

The power of the methods under causal dosage × length effects in TGP-WG is shown in S2 Fig. We observe that CONCUR has the best or comparable power with the second best method (CCRET) across different patterns of deleterious-protective effects and in the duplication, deletion, and combined effects scenarios. Both CONCUR and CKAT are designed to detect dosage × length signals, but CKAT struggled to pick up the signals. One possible reason might be the multiple testing penalty applied to CKAT. In addition, the CNV signals in these simulations originate from aligned genomic regions; such signals may or may not be well-captured by CKAT, since its scanning algorithm may incorporate CNV similarity from off-position CNV events or same-position CNV events dependent on the data.

We note that we do not expect the methods to display similar relative performance in the duplication-only causal effects and deletion-only causal effects scenarios. This is because the causal segments for duplications versus deletions have different characteristics, due to differences in the patterns of duplications overlapping versus deletions overlapping in the data. In addition, the strength of the signal in the duplication-only and deletion-only simulations differs due to differences in the length of the segments as well as the frequency of CNVs in the protective versus deleterious segments. For example, in the duplication-only effects (D,P) = (10,90) setting, the combination of longer segments and more CNV activity in the protective segments leads to a proportionally larger protective signal than in the corresponding deletion-only setting. The result of these differences is asymmetry in the methods’ performance in the two settings.

The power under causal dosage effects is shown in S3 Fig. As expected, the dosage-based CCRET kernel performs the best, with CONCUR following CCRET or having comparable power.

#### Results of TGP-Chr1 simulations

**Fig 2:**
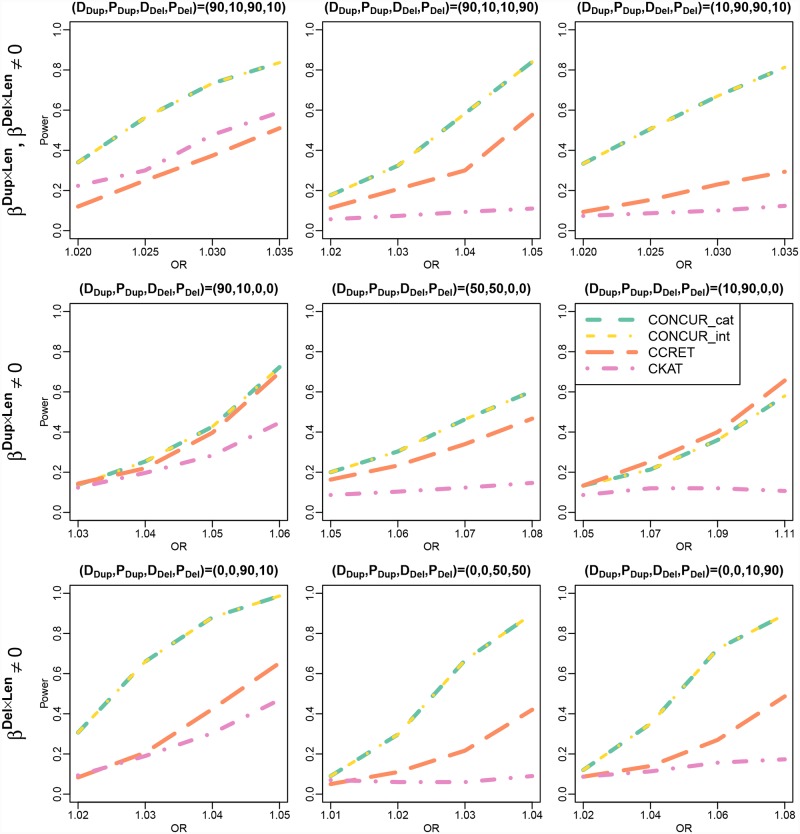
Power comparison between CONCUR, CCRET, and CKAT in the TGP-Chr1(a) simulations (causal dosage × length effects in chromosome 1 of TGP data). The top panel shows power under combined duplication and deletion effects, the middle panel shows power under effects from duplications only, and the bottom panel shows power under effects from deletions only. Different proportions of deleterious vs. protective effects are considered as indicated by (D_Dup_,P_Dup_,D_Del_,P_Del_) with D_Dup_ and P_Dup_ reflecting the proportions of deleterious and protective segments among causal duplication segments, and with D_Del_ and P_Del_ defined similarly for causal deletion segments.

The results of the TGP-Chr1(a) simulations are shown in Fig 2. We observe that CONCUR has higher than or comparable power to the second best method, and here the second best method is CKAT or CCRET depending on the scenario. After further exploration of the TGP-Chr1(a) simulations as well as TWB-Chr1 dosage × length simulations, it appears that the relative performance between CKAT and CCRET depends more heavily on whether the causal signals can be well-captured by the CKAT kernel than what the patterns of causal effects are (i.e., causal effects from duplications, deletions, or both, or the proportion of deleterious vs. protective effects). The power from CONCUR_cat and CONCUR_int appear to be identical in all settings, which is not surprising given that there are very few CNVs of larger magnitude (0 or 4+) in the data.

**Fig 3:**
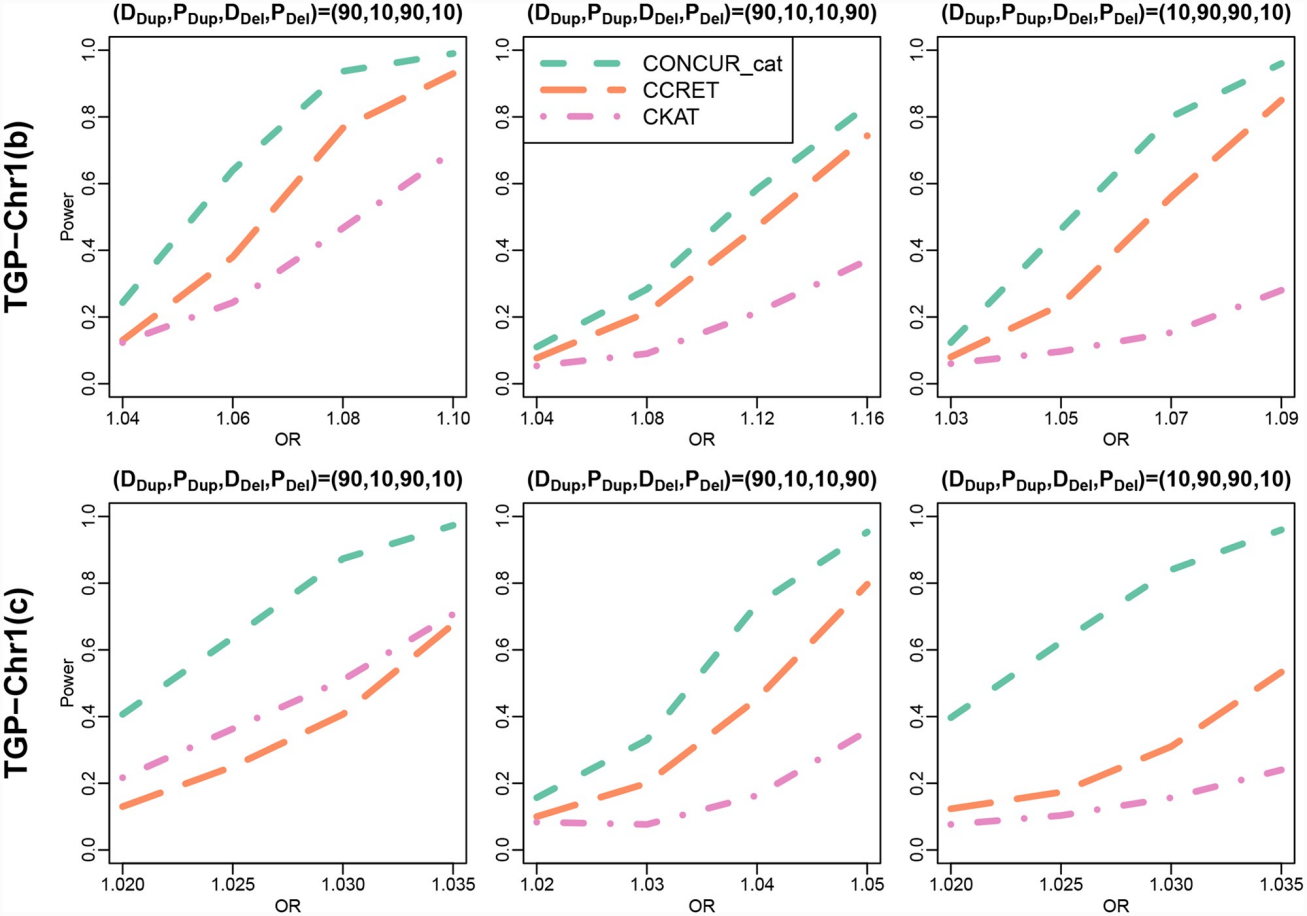
Power comparison between CONCUR, CCRET, and CKAT in TGP-Chr1(b) and TGP-Chr1(c) (causal dosage × length effects from both duplications and deletions in chromosome 1 of TGP data). The top panel shows power under TGP-Chr1(b), in which the kernels are built on CNV data with error added to the CNV endpoints, mimicking the scenario of inaccuracy end points of called CNVs. The bottom panel shows power under TGP-Chr1(c), in which the disease base rate is more rare (5%). Different proportions of deleterious vs. protective effects are considered as indicated by (D_Dup_,P_Dup_,D_Del_,P_Del_) with D_Dup_ and P_Dup_ reflecting the proportions of deleterious and protective segments among causal duplication segments, and with D_Del_ and P_Del_ defined similarly for causal deletion segments.

Fig 3 shows the performance of the methods applied to data with inaccurate CNV endpoint information (top panel) and under a lower baseline disease rate (5%; lower panel). For both analyses, the top row of Fig 2 is a useful reference showing the methods’ performance under error-free CNV data and a higher baseline disease rate (12%). The top panel of Fig 3 shows that CONCUR still has higher power than the baseline methods, although the gap between CONCUR and CCRET is smaller compared to the error-free scenario. The lower panel of Fig 3 demonstrates that the performance of the methods under a lower baseline disease rate is very comparable to that under a higher disease rate.

#### Results of TWB-Chr1 simulations

**Fig 4:**
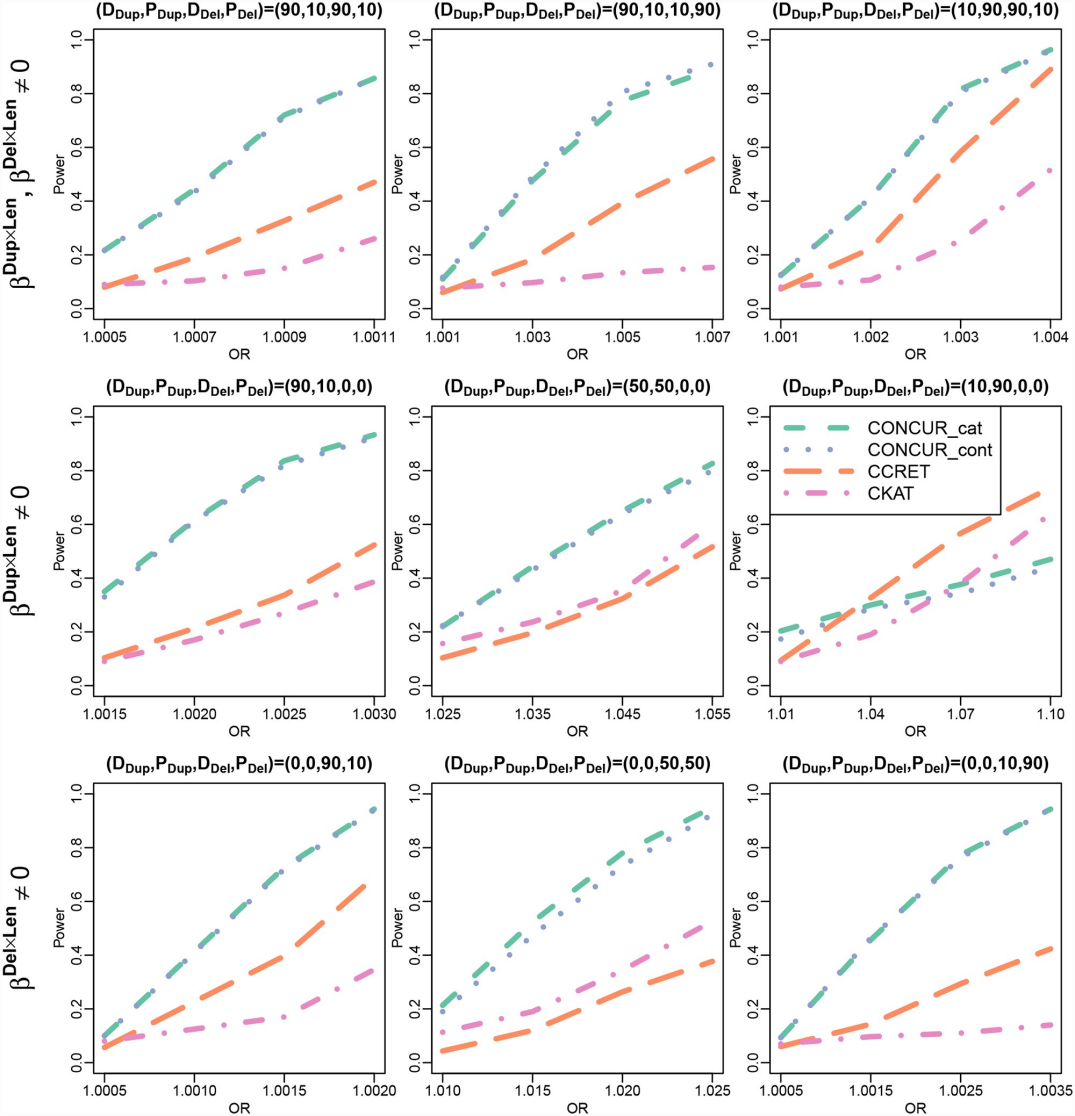
Power comparison between CONCUR, CCRET, and CKAT in TWB-Chr1(a) (causal continuous dosage × length effects in chromosome 1 of TWB data). The top panel shows power under combined duplication and deletion effects, the middle panel shows power under effects from duplications only, and the bottom panel shows power under effects from deletions only. Different proportions of deleterious vs. protective effects are considered as indicated by (D_Dup_,P_Dup_,D_Del_,P_Del_) with D_Dup_ and P_Dup_ reflecting the proportions of deleterious and protective segments among causal duplication segments, and with D_Del_ and P_Del_ defined similarly for causal deletion segments.

The results of TWB-Chr1(a) are shown in Fig 4. We observe little difference in the power of the two CONCUR approaches. We also observe that CONCUR had stronger power than CCRET and CKAT, with the exception of some degenerate behavior in CONCUR under (D_Dup_, P_Dup_, D_Del_, P_Del_) = (10, 90, 0, 0). In this setting, we suspect that CONCUR’s behavior is due to the combination of a couple factors. There are few duplication events to begin with in the TWB simulated data, and that combined with the 90% protective effects leads to the simulated controls being comprised primarily of “random” controls with relatively few individuals carrying causal protective CNVs. This results in an extremely weak signal-to-noise ratio. All methods were affected in this setting, such that we needed to significantly boost the range of effect sizes to observe power in any method (e.g., 1.01-1.10 here vs. 1.0005-1.0035 under (D_Dup_, P_Dup_, D_Del_, P_Del_) = (0, 0, 10, 90)). We believe that CCRET and CKAT are more robust in this setting due to borrowing information across loci through CNVRs and through across-position alignment, respectively. Finally, as in the TGP-Chr1 simulations, under some scenarios CKAT had higher power than CCRET, e.g., in the settings of (D_Dup_, P_Dup_, D_Del_, P_Del_) = (50, 50, 0, 0) and (0, 0, 50, 50). The relative performance of the two methods is again likely dependent on whether the causal signals were well captured by the CKAT kernel.

**Fig 5:**
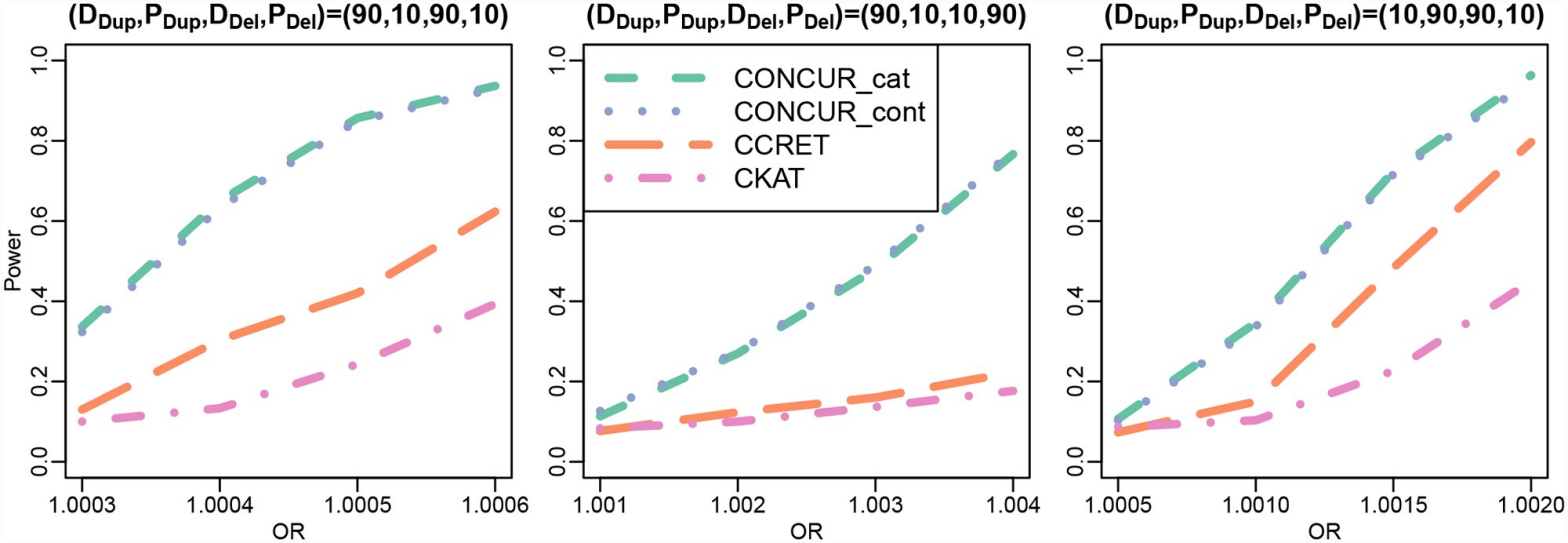
Power comparison between CONCUR, CCRET, and CKAT for TWB-Chr1(b) (causal categorical dosage × length effects in chromosome 1 of TWB data). The panels show power under combined duplication and deletion effects. Different proportions of deleterious vs. protective effects are considered as indicated by (D_Dup_,P_Dup_,D_Del_,P_Del_) with D_Dup_ and P_Dup_ reflecting the proportions of deleterious and protective segments among causal duplication segments, and with D_Del_ and P_Del_ defined similarly for causal deletion segments.

Fig 5 evaluates the ability of the CONCUR_cont approach to detect a signal from categorical dosages, on which CONCUR_cat, CCRET, and CKAT are built. We observe nearly identical power in CONCUR_cont and CONCUR_cat.

### Real data application

In real data applications, we first conducted CNV association tests on a previously analyzed CNV dataset from the Swedish Schizophrenia Study as a proof of concept. We next conducted a CNV-triglyceride (TG) association analysis on data from the Taiwan Biobank. We constructed kernels for all methods (CONCUR, CKAT and CCRET) based on categorical dosages, and dropped the “_cat” suffix in our discussion of the CONCUR method in this section for simplicity. We used the step-wise Holm method to adjust for multiple testing, which yields more powerful results than the Bonferroni procedure and remains valid when applied to dependent p-values [14].

#### CNV analysis on schizophrenia in the Swedish Schizophrenia Study

We conducted pathway-based CNV analysis on data from the Swedish Schizophrenia Study [15]. The Swedish Schizophrenia Study used a case-control sampling design. Genotyping was done in six batches using Affymetrix 5.0 (3.9% of the subjects), Affymetrix 6.0 (38.6%), and Illumina OmniExpress (57.4%). PennCNV [11] was used to generate CNV calls. After quality control, we obtained a high quality rare CNV (frequency < 1% and size > 100kb) dataset in 8,457 subjects (3,637 cases and 4,820 controls) [16]. Previous analyses of this data [16] indicated significant associations of large rare CNVs with schizophrenia risk for both genome-wide dosage effects and gene intersecting effects of selected gene sets.

To evaluate the practical utility of the three kernel-based tests, we performed analysis on the gene sets previously examined in [6], excluding the PSD pathway as it overlaps the other three PSD-related pathways considered. In the 8 gene sets, large (> 500kb) rare CNVs were found to be associated with schizophrenia by Szatkiewicz et al. [16], and these associations were corroborated by Tzeng et al. [6] in a gene-interruption analysis with CNVs > 100kb. In each pathway analysis, we performed association tests for joint dosage and length effects of rare CNVs > 100kb, using a fixed effect term to adjust for batch effects. CONCUR and CKAT kernels were constructed from the raw PLINK data and the CCRET dosage kernel was created using the functions available on the CCRET website. For CKAT, we used pathways as the CNVR unit instead of chromosomes because there were multiple chromosomes with only one gene.

After applying Holm’s procedure to adjust for multiple testing on the 8 pathways, CONCUR and CKAT found significant associations in all 8 pathways, while CCRET identified 4 pathways as significant (Table 2). We observed stronger power in CKAT in the analysis here compared to the power observed in the simulation studies. CKAT and CONCUR are more sensitive to dosage-length effects while CCRET is more sensitive to dosage effects; thus, these results suggest significant CNV effects from dosage × length or length affecting schizophrenia risk in several pathways.

**Table 2:** Association test results for the effects of CNVs with > 100kb in length on schizophrenia risk in the Swedish Schizophrenia Study. Raw p-values are reported for CONCUR, CCRET, and CKAT. Asterisks indicate p-values that were significant after a Holm multiple-testing adjustment. Pathways are ordered alphabetically.

	Gene-sets			P-values		
Gene-set Name	# Genes	# Genes Interrupted in Cases	# Genes Interrupted in Controls	*CONCUR*	*CCRET*	*CKAT*
Cytoplasm (Kirov et al. [17])	266	28	32	0.00124^⋆^	0.01408	0.00030^⋆^
FMRP targets (Darnell et al. [18])	810	149	152	2.29E-05^⋆^	0.00044^⋆^	0.00026^⋆^
Mental Retardation	503	67	63	0.00164^⋆^	0.10200	0.00350^⋆^
PSD/mGluR5 (Kirov et al. [17])	38	4	7	0.00040^⋆^	0.10540	0.00129^⋆^
PSD/NMDAR (Kirov et al. [17])	61	12	12	0.00102^⋆^	0.00922^⋆^	0.00046^⋆^
PSD/PSD-95 (Kirov et al. [17])	65	13	10	0.00052^⋆^	0.00144^⋆^	0.00903^⋆^
Synaptic genes (Ruano et al. [19])	718	154	164	5.45E-06^⋆^	0.02005	0.00766^⋆^
Synaptic Proteome (G2Cdb)	1023	121	106	0.00067^⋆^	0.00010^⋆^	0.00736^⋆^

#### CNV analysis on triglycerides in the Taiwan Biobank

We applied the proposed CONCUR test to the Taiwan Biobank (TWB) data https://www.twbiobank.org.tw/new_web/ and conducted CNV association analysis with triglyceride (TG) levels on lipid-related pathways. The nationwide biobank project was initiated in 2012 and has recruited more than 15,995 individuals. Peripheral blood specimens were extracted from healthy donors and genotyped using the Affymetrix Genomewide Axiom TWB array, which was designed specifically for a Taiwanese population. The TWB array contains 653,291 SNPs and was used to generate calls for genome-wide CNVs in the following process. First, Affymetrix Power Tools version 1.18.0 was used to produce a summary file of the intensity values of all probes, and the file was input into the Partek Genomic Suite version 6.6 to call CNVs based on the following criteria: at least 35 consecutive SNP markers, p-values of different CN values between two consecutive segments < 0.001, and signal-to-noise ratio (SNR) ≥ 0.3. A duplication was called if its copy number was ≥ 2.3, and a deletion was called if its copy number was ≤ 1.7. Several previous studies [20] [21] have demonstrated appropriate CNV calls with these parameters. After quality control, we obtained CNV data in 14,595 unrelated individuals. Our CNV association analyses focused on a subset of 11,664 individuals who had non-missing TG levels.

We referenced the Kyoto Encyclopedia of Genes and Genomes (KEGG) pathway database [22] to identify lipid-related pathways. Among the 17 pathways related to “lipid metabolism”, 15 pathways included genes intersected by the TWB CNV data and were selected as candidate pathways in our analysis. For each pathway analysis, we adjusted for sex, age, BMI, and the top 10 principal components representing the population structure as covariates with fixed effects. As before, CKAT was implemented with each pathway comprising a single CNVR.

Similar to the Swedish Schizophrenia Study analysis, after applying Holm’s procedure to adjust for multiple testing on the 15 pathways, all 15 pathways were found significant by CONCUR and CKAT, and 1 pathway was found significant by CCRET (Table 3). CKAT again demonstrated much better power than in the simulation studies. The relative performance among the three methods may be due to more dominant length or dosage × length signals.

**Table 3:** Association test results for the effects of CNVs on triglyceride levels in the Taiwan Biobank. Raw p-values are reported for CONCUR, CCRET, CKAT, and a negative control test in which CONCUR is applied to a randomly permuted response vector. Asterisks indicate p-values that were significant after a Holm multiple-testing adjustment. Pathways are ordered alphabetically.

Gene-sets			P-values			
Gene-set Names	# Genes	# Genes Interrupted	*CONCUR*	*CCRET*	*CKAT*	Neg. Control
hsa00061 (Fatty acid biosynthesis)	13	12	0.00171^⋆^	0.01187	0.00197^⋆^	0.89046
hsa00062 (Fatty acid biosynthesis)	30	26	0.00031^⋆^	0.01591	0.00508^⋆^	0.36231
hsa00071 (Fatty acid degradation)	44	43	0.00406^⋆^	0.01088	0.00631^⋆^	0.78391
hsa00072 (Synthesis and degradation of ketone bodies)	10	10	0.00008^⋆^	0.00459	0.00383^⋆^	0.37588
hsa00100 (Steroid biosynthesis)	19	16	0.01641^⋆^	0.00618	0.00906^⋆^	0.28308
hsa00120 (Primary acid bile biosynthesis)	17	17	0.00019^⋆^	0.00314^⋆^	0.00274^⋆^	0.56475
hsa00140 (Steroid hormone biosynthesis)	60	58	0.00030^⋆^	0.00623	0.00159^⋆^	0.25125
hsa00561 (Glycerolipid metabolism)	61	50	0.00430^⋆^	0.00494	0.00198^⋆^	0.46773
hsa00564 (Glycerophospholipid metabolism)	97	86	0.00322^⋆^	0.00398	0.00209^⋆^	0.52954
hsa00565 (Ether lipid metabolism)	47	43	0.00018^⋆^	0.00859	0.00439^⋆^	0.50786
hsa00590 (Arachnidonic acid metabolism)	63	62	0.00212^⋆^	0.00883	0.00211^⋆^	0.78048
hsa00591 (Linoleic acid metabolism)	29	29	0.00080^⋆^	0.01799	0.00291^⋆^	0.65068
hsa00592 (alpha-Linolenic acid metabolism)	25	25	0.00581^⋆^	0.00927	0.00273^⋆^	0.74765
hsa00600 (Sphingolipid metabolism)	47	43	0.00382^⋆^	0.00512	0.00789^⋆^	0.31366
hsa01040 (Biosynthesis of unsaturated fatty acids)	27	23	0.00012^⋆^	0.00394	0.00158^⋆^	0.77243

Next, we explored associations with TG levels in the TWB data on a by-chromosome basis (Table 4). Using the Holm method to adjust for multiple testing on 22 chromosomes, CONCUR found all 22 chromosomes significantly associated with TG, CKAT found 12 significant chromosomes, and CCRET found none. It is not unexpected to see all 22 chromosomes identified to be significantly associated with TG, since the genes in the 15 significant lipid metabolism pathways examined are located across all 22 chromosomes. For example, for the 12 chromosomes identified by both CONCUR and CKAT, the number of pathway genes intersected by CNVs ranges from 8 to 41, and for those chromosomes uniquely identified by CONCUR (excluding chromosome 13), the number of intersected genes ranges from 4 to 36. In chromosome 13, while there is only one pathway gene, all CNVs are located in chr13q, which is a well-studied region related to cholesterol metabolism [23] [24] [25]. Since cholesterol is strongly related to TG levels, CNVs in chr13q and chr13q22-q32 may impact TG levels by affecting the metabolism efficiency of TG and cholesterol. To further interpret the significant CONCUR test result, we examined the subregion chr13q22-q32 that is highlighted in [24] and contains or overlaps with the markers in [23] and [25]. By applying CONCUR, CKAT and CCRET to this subregion, we obtained the p-values as 0.0000143, 0.0004388 and 0.0242815, respectively. These results suggest a length or dosage × length signal arising from chr13q22-q32, which CONCUR and CKAT can detect with good power. This length-driven CNV signal is not well captured by CCRET in both of the subregion and chromosome-wide analyses, since CCRET does not account for CNV length features. The strength of the signal from chr13q22-q32 may be diluted for CKAT when the entire chromosome is treated as a CNVR.

**Table 4:** Association test results for the effects of CNVs on triglyceride levels by chromosome in the Taiwan Biobank. Results from the CONCUR, CCRET, and CKAT association tests are shown. The results of the negative control analysis reflect the p-value from CONCUR applied to a randomly permuted response vector. Asterisks indicate p-values that were significant after a Holm multiple-testing adjustment. Pathways are ordered according to chromosome. For interpretation of the by-chromosome association tests, the number of genes from the 15 lipid metabolism pathways that are intersected by CNVs is given (# Genes Interrupted).

Chromosomes		P-values			
Chr	# Genes Interrupted	*CONCUR*	*CCRET*	*CKAT*	Neg. Control
1	41	0.000018^⋆^	0.734017	0.000017^⋆^	0.739204
2	30	0.000007^⋆^	0.077438	0.000118^⋆^	0.790286
3	14	4.41E-08^⋆^	0.363502	0.000834^⋆^	0.483097
4	36	5.11E-08^⋆^	0.257951	0.035544	0.900595
5	12	1.25E-07^⋆^	0.153555	0.003703^⋆^	0.865209
6	18	4.00E-07^⋆^	0.080112	0.000105^⋆^	0.735159
7	13	0.000030^⋆^	0.216259	0.001868^⋆^	0.855037
8	14	0.000006^⋆^	0.627055	0.000229^⋆^	0.781897
9	15	0.000663^⋆^	0.109319	0.006223	0.962761
10	29	0.000082^⋆^	0.256622	0.000027^⋆^	0.603895
11	20	0.000034^⋆^	0.002463	0.000557^⋆^	0.623104
12	15	0.000006^⋆^	0.316674	0.009471	0.484621
13	1	0.000010^⋆^	0.195530	0.217677	0.883870
14	10	0.000070^⋆^	0.075694	0.000149^⋆^	0.608107
15	14	0.000263^⋆^	0.464109	0.000635^⋆^	0.768470
16	11	0.001748^⋆^	0.217819	0.008337	0.604125
17	22	0.001328^⋆^	0.024979	0.015718	0.658522
18	5	0.000009^⋆^	0.155285	0.017809	0.954988
19	21	0.012431^⋆^	0.514188	0.009205	0.663204
20	8	0.000079^⋆^	0.120546	0.001250^⋆^	0.888174
21	4	0.000511^⋆^	0.200590	0.005763	0.583767
22	13	0.011617^⋆^	0.946787	0.019274	0.786773

As further assurance that these associations are less likely due to false positives, we conducted a CONCUR negative-control analysis by repeating the by-chromosome analysis using permuted TG levels. The resulting p-values are shown in Table 4; the p-value range of those chromosomes identified by both CONCUR and CKAT (i.e., 0.483 to 0.888) is similar to that of those uniquely identified by CONCUR (i.e., 0.485 to 0.963). In addition, we also examined the quantile-quantile plots (Q-Q plots) of CONCUR p-values from negative-control analyses, by generating (a) 20 TG-permuted datasets for each of the 15 pathways, and (b) 1000 TG-permuted datasets for chromosome 13. The Q-Q plots, shown in S4 Fig, suggest that the p-values follow the expected null distribution, Uniform(0,1).

Finally, we illustrate in S3 Appendix possible CONCUR post-hoc analyses probing the potential sources of a CNV association identified at the aggregate-level. As an example, we looked more closely at pathway hsa01040 (biosynthesis of unsaturated fatty acids), for which both CONCUR and CKAT were significant but not CCRET. In short, we calculated summary statistics describing CNV length and dosage in hsa01040 for individuals with different levels of TG (low, medium, and high), and examined CNV features in all CNVs together and in duplications and deletions separately. We also used heatmaps to visualize CNVs in the 23 genes in hsa01040 (Fig A in S3 Appendix), displaying the duplications and deletions intersecting genes in all CNV profiles categorized by their TG level. These exploratory analyses suggested that for duplications only, there may exist “promising” differences in CNV length and relatively weaker differences in dosage across TG levels. Because these “promising” associations from a stratified analysis reflected only marginal associations of a CNV feature and did not account for the effect heterogeneity that motivates the application of kernel-based methods, we also applied CONCUR to duplications and deletions separately, and found a very significant association with TG in duplications (p-value < 1 × 10^−8^) and a weaker signal in deletions (p-value = 0.0313).

## Discussion

We introduce CONCUR to leverage the strength of kernel-based methods to assess the collective effects of rare CNVs on disease risk and incorporate several desired features. First, CONCUR permits the quantification of CNV similarity in an CNVR-free manner, avoiding the need of arbitrarily defining CNVRs as in current practice. Second, CONCUR incorporates both length and dosage information via the cAUC kernel, and is capable of detecting dosage, length and length-dosage interaction effects. Third, as the technology for detecting smaller CNVs improves, we expect to observe more length variation in CNVs and an increasing need to accommodate length effects in CNV association studies. However, there are shortcomings in the standard kernel choices for handling CNV length. For example, a linear (or polynomial) kernel, which scores length similarity in a multiplicative fashion, cannot always reflect the true level of length similarity between an individual pair: e.g., a pair of CNVs of length 20 would be equally similar to two CNVs with lengths 1kb and 400kb (as 20 × 20 = 1 × 400). The alternative Gaussian kernel as in CKAT would still require a pre-specified scaling factor. CONCUR addresses these issues by using the common AUC of the CN profile curves of an individual pair, quantifying CNV similarity in dosage and length simultaneously. Finally, unlike current kernel methods which require discretized copy numbers, CONCUR is directly applicable to continuous and discrete copy numbers. We implement the CONCUR test in the R package ‘CONCUR’.

CONCUR shares some philosophy with several CNV analysis strategies in the literature. For example, Aguirre et al. [26] characterized the copy number changes in the pancreatic adenocarcinoma genome by detecting the minimum common regions (MCR) of recurrent copy number changes across tumor samples and using MCRs to prioritize genes that might be involved in pancreatic carcinogenesis. Harada et al. [27] also examined the minimal overlapping/common regions of frequent CNV activities among pancreatic cancer samples and among normal samples to identify candidate regions that might contain critical oncogenes or tumor suppressor genes. Furthermore, Mei et al. [28] proposed algorithms for identifying common CNV regions across individuals of homogeneous phenotypes for downstream association analysis. Built on similar concepts to these “common regions”, CONCUR quantifies CNV similarity between sample pairs based on the “size” of the common regions as reflected in congruent location and dosage, and provides an association test to evaluate dosage and length effects.

In the analyses performed in this study, we calculated the cAUC using CNV dosage values transformed by the functions *a*^*Dup*^(*d*) = |*d* − 2| for duplications and 0 otherwise, and *a*^*Del*^(*d*) = |*d* − 2| for deletions and 0 otherwise. That is, we used copy number 2 as a reference value, and defined CNV similarity as the overlapping CNV length scaled linearly according to the magnitude of dosage deviation from the reference value. As indicated in the method section, CONCUR can be flexibly extended to accommodate other schemes of quantifying common area by adopting different *a*^•^(⋅) functions in the calculation of the cAUC, e.g., *a*^•^(*d*) = |*d* − 2|^*c*^ with *c* ≠ 1. Finally, overlapping area may be further weighted by inverse frequencies or according to CNV type (e.g., deletion) when needed, to augment the contribution from overlapping regions from rarer CNVs or from CNVs with more severe impact, respectively.

We note that although both CONCUR and CKAT are designed to capture CNV dosage and length information, the two kernels are constructed based on different philosophies, and each method has sensitivity to certain effect mechanisms. CONCUR first quantifies the similarity in an individual pair within the same genomic locations, and then collapses the similarity information across different locations in a user-specified region (e.g., whole genome, chromosome, or gene set). In contrast, CKAT treats the user-specified region as a CNVR, and collapses CNV information across different locations by incorporating off-position similarities and/or same-position similarities to obtain a CNVR-level measure of similarity.

The different philosophies of quantifying CNV similarity may also explain the differences in CKAT’s relative performance compared to CCRET here versus in the CKAT paper [7]. Here we observe that CKAT has higher or comparable power compared to CCRET in some scenarios and lower power in other scenarios; however, in the CKAT paper, CKAT outperforms CCRET in the majority of the considered scenarios. This discrepancy is likely due to differences in the assumed effect mechanisms in our approach versus those in the CKAT paper. In the simulation study here, certain genomic regions are selected to be causal. Whereas, in the CKAT paper simulations, causal effects arise from CNVs, not genomic regions. Each individual has either 0 or 1 CNV with randomly generated endpoints; CNVs of the same type (i.e., duplication or deletion) are either all causal or all non-causal, depending on the scenario. Under this design, causal CNVs in different individuals may fall in different genomic locations, yet CNVs of the same type will have similar effects. CKAT powerfully detects these signals because its similarity quantification approach (i.e., based on pairs of CNVs rather than aligned CNV activity in a fixed genomic location) better captures the CNV-driven (as opposed to locus-driven) signal. Whereas, methods that quantify similarity based on aligned positions, such as CCRET and CONCUR, can suffer from power loss under this signal, as exemplified by the performance of CCRET in [7].

## Materials and methods

### Ethics statement

In the Swedish Schizophrenia Study, all procedures were approved by ethical committees at the Karolinska Institutet (Dnr No. 04/-449/4 and No. 2015/2081-31/2) and University of North Carolina (No. 04-1465 and No. 18-1938). All subjects provided written informed consent (or legal guardian consent and subject assent). The Taiwan Biobank study was approved by the ethical committee at Taichung Veterans General Hospital (IRB TCVGH No. CE16270B-2). Consent was not obtained because the data were de-identified.

### CONCUR method

For individual *i*, *i* = 1, ⋯, *n*, denote *Y*_*i*_ the phenotype of individual *i*. Codify the CNV information in matrix *Z*_*i*_ with dimension *P*_*i*_ × 4 as in the standard PLINK format of CNV data, where *P*_*i*_ is the number of CNVs that individual *i* has, and each row of *Z*_*i*_ records four features of CNV *p*, *p* = 1, ⋯, *P*_*i*_: dosage (denoted as *d*_*p*_) chromosome (denoted as *Chr*_*p*_), start location (denoted as *BP*1_*p*_), and end location (denoted as *BP*2_*p*_). The dosage *d*_*p*_ can be integer, continuous or categorical values. For example, in the Swedish Schizophrenia pathway analysis, an individual might have between 1-88 CNVs, and CNV lengths might range from 100kb up to 7841kb. Let *X*_*i*_ = (*X*_*i*1_, ⋯, *X*_*ir*_)^*T*^ be the *r* covariates. Under the kernel machine regression framework, we model the association between phenotypes and CNVs as follows

$$\begin{array}{c} g\left(\mu_{i}\right)=\beta_{0}+X_{i}^{T}\beta_{X}+h\left(Z_{i}\right), \end{array}$$ (2)

where *μ*_*i*_ = *E*(*Y*_*i*_|*X*_*i*_, *Z*_*i*_), the conditional phenotype mean given covariates and CNVs; *g*(⋅) is the canonical link, which transforms the conditional phenotype mean *μ*_*i*_ so that the mean is on the same scale of the linear predictors formed by covariates and CNV data. For continuous phenotypes, *g*(*μ*_*i*_) = *μ*_*i*_; for binary phenotypes, *g*(*μ*_*i*_) = log[(*μ*_*i*_/(1 − *μ*_*i*_)]. *h*(*Z*_*i*_) is an unknown smooth function of the variant features characterized by a kernel function *k*(⋅, ⋅).

#### Profile curves

The proposed cAUC kernel is built on the concept of a CN profile curve as shown in Fig 1. Consider the genomic location *x* from chromosome *k* for individual *i*. Given the CN profile curve, we define the duplication profile curve, $f_{ik}^{Dup}\left(x\right)$, and the deletion profile curve, $f_{ik}^{Del}\left(x\right)$, which recenter and rescale the CN values in CN profile curves through the “dosage transform functions” as described below, and allow us to compute cAUC similarity from duplications and from deletions in a more flexible manner. Specifically, let *q* = 1, ⋯, *P*_*ik*_ index the CNV features (*d*_*q*_, *BP*1_*q*_, *BP*2_*q*_) occurring on chromosome *k* of individual *i*. Then we construct duplication and deletion profile curves respectively describing duplications and deletions on chromosome *k* for individual *i* as follows:

$$\begin{array}{cc} f_{ik}^{Dup}\left(x\right) & =\sum_{q=1}^{P_{ik}}I\left(BP1_{q}\leq x\leq BP2_{q}\right)a^{Dup}\left(d_{q}\right) \end{array}$$ (3)

$$\begin{array}{cc} f_{ik}^{Del}\left(x\right) & =\sum_{q=1}^{P_{ik}}I\left(BP1_{q}\leq x\leq BP2_{q}\right)a^{Del}\left(d_{q}\right) \end{array}$$ (4)

where *x* is a location on the genome on the same scale as *BP*1_*q*_ and *BP*2_*q*_; *I* is the indicator function such that *I*(⋅) = 1 if the condition contained within is satisfied and equals 0 if otherwise; and *a*^•^(*d*) is a dosage transform function which determines the reference copy number value and controls how different copy number values contribute more or less to similarity in profiles. If an individual has no CNVs in chromosome *k*, then their duplication and deletion profile curves are identically equal to zero, i.e., $f_{ik}^{Dup}\left(x\right)=f_{ik}^{Del}\left(x\right)\equiv 0$ for all *x*. Although not explicitly shown, $f_{ik}^{Dup}$ and $f_{ik}^{Del}$ are functions of *Z*_*i*_ as the information of *d*_*q*_, *BP*1_*q*_, *BP*2_*q*_ and chromosome *k* for subject *i* is obtained from *Z*_*i*_.

In this study, we designated *a*^*Dup*^(*d*_*q*_) = |*d*_*q*_ − 2| if *d*_*q*_ is from a duplication and 0 otherwise. That is, for a given chromosome *k* and individual *i*, the function $f_{ik}^{Dup}\left(x\right)$ equals the magnitude of the duplication (i.e., number of additional copies compared to the reference copy number 2) for *x* inside a duplication and equals 0 otherwise. For deletions, *a*^*Del*^(*d*_*q*_) and $f_{ik}^{Del}\left(d_{q}\right)$ can be obtained in an analogous way.

#### cAUC kernel

We propose to quantify the similarity between individuals *i* and *j* by comparing $f_{ik}^{Dup}$ vs. $f_{jk}^{Dup}$ and $f_{ik}^{Del}$ vs. $f_{jk}^{Del}$ over chromosomes *k* = 1, ⋯, 22 using the following kernel function:

$$\begin{array}{c} k_{cAUC}\left(Z_{i},Z_{j}\right)=\sum_{k=1}^{22}\int_{N}[\min(f_{ik}^{Dup}\left(x\right),f_{jk}^{Dup}\left(x\right))+\min(f_{ik}^{Del}\left(x\right),f_{jk}^{Del}\left(x\right))]\text{d}\mu \left(x\right) \end{array}$$ (5)

where $\min(f_{ik}^{•}\left(x\right),f_{jk}^{•}\left(x\right))$ captures the minimum of the two functions evaluated at *x* and *μ*(*x*) is the counting measure. We refer to the kernel function as the cAUC kernel as it computes the minimal common area under the two individuals’ duplication and deletion profile curves. The cAUC kernel matrix **K**_*cAUC*_ is constructed such that its (*i*, *j*)th element is *k*_*cAUC*_(*Z*_*i*_, *Z*_*j*_). The cAUC kernel is a valid kernel as shown in S1 Appendix.

As an illustrating example, we calculate the cAUC between individuals 1 and 2 from Fig 1. Both individuals have duplication profile curves on chromosome 1 as $f_{11}^{Dup}\left(x\right)=f_{21}^{Dup}\left(x\right)=0$, since they have no duplications. For individual 1, the deletion profile curve is $f_{11}^{Del}\left(x\right)=\left|d-2\right|=\left|0-2\right|=2$ if *x* ∈ [200, 400] on chromosome 1 and 0 otherwise; for individual 2, $f_{21}^{Del}\left(x\right)=2$ if *x* ∈ [100, 500], 1 if *x* ∈ [600, 800], and 0 otherwise. To compute their cAUC, we characterize the individuals’ curves in 2 genomic regions: (1) *x* ∈ [200, 400], in which $f_{11}^{Del}\left(x\right)=f_{21}^{Del}\left(x\right)=2$; and (2) *x* ∉ [200, 400], in which $\min[f_{11}^{Del}\left(x\right),f_{21}^{Del}\left(x\right)]=0$. This discretization allows us to compute the cAUC by multiplying the minimum value of the two curves in each region by the length of the region to obtain *k*_*cAUC*_(*Z*_1_, *Z*_2_) = (2 × 200) + 0 = 400.

The intuition of the cAUC kernel is to quantify similarity using the length of overlapping CNVs between two individuals, with dosage information of the two overlapping CNVs determining how the overlapping length is scaled. The minimum operator enforces that the overlapping length is scaled by the CNV of smaller magnitude in a pair with different magnitudes. The similarity between CNVs of different types (i.e., duplication vs. deletion) is 0. The similarity between CNVs of the same type depends on the copy number values via the dosage transform function, *a*^•^(*d*). Legal choices of *a*^•^(*d*) will upweight the contribution from similar CNVs of greater magnitude in duplication or deletion, which are often more rare and have higher impact. The family of dosage transform functions *a*^•^(*d*) = |*d* − 2|^*c*^ provides a spectrum of weighting schemes, with *c* < 1 down-weighting and *c* > 1 upweighting the contribution of higher magnitude CNVs. Across copy number data of varying types and varying sample-level characteristics, the *a*^•^(⋅) dosage transform function allows for flexible scaling of dosage to appropriately customize the cAUC measure of similarity.

#### Association test

The association between phenotype and CNVs is examined by testing the hypothesis *H*_0_: *h*(⋅) = 0. To do so, we define the vector of subject-specific CNV effects *H* = (*h*(*Z*_1_), ⋯, *h*(*Z*_*n*_)) and treat *H* as random effects which follow *N*(0, *τ***K**), where *τ* ≥ 0 is a variance component and **K** is a *n* × *n* kernel matrix with its (*i*, *j*)th entry being *k*(*Z*_*i*_, *Z*_*j*_). Following Liu et al. [29] [30], testing *H*_0_: *h*(⋅) = 0 is equivalent to testing *τ* = 0 under a generalized linear mixed model. As in [6] [7], we use a score-based test, which is

$$\begin{array}{c} T=\frac{1}{2{\hat{\sigma }}^{2}}{\left(Y-{\hat{\mu }}_{0}\right)}^{T}K\left(Y-{\hat{\mu }}_{0}\right) \end{array}$$ (6)

for continuous phenotypes, and is

$$\begin{array}{c} T=\frac{1}{2}{\left(Y-{\hat{\mu }}_{0}\right)}^{T}K\left(Y-{\hat{\mu }}_{0}\right) \end{array}$$ (7)

for binary phenotypes. In the score statistic *T*, *Y* = (*Y*_1_, ⋯, *Y*_*n*_)^*T*^ and *σ*^2^ is the variance of the continuous *Y*. Define *μ* = (*μ*_1_, ⋯, *μ*_*n*_)^*T*^ with *i*-th element *μ*_*i*_ = *E*(*Y*∣*X*_*i*_, *Z*_*i*_); then ${\hat{\mu }}_{0}$ is the estimate of *μ* in Eq 2 under *H*_0_: *h*(⋅) = 0. Specifically, ${\hat{\mu }}_{0}={\hat{\beta }}_{0}+X{\hat{\beta }}_{X}$ for continuous phenotypes and ${\hat{\mu }}_{0}={\text{logit}}^{-1}({\hat{\beta }}_{0}+X{\hat{\beta }}_{X})$ for binary phenotypes. The score statistic asymptotically follows a weighted chi-square distribution [29] [30]. Recently, Chen et al. [12] derived the corresponding small-sample distribution, which is used to calculate the p-value in this work.

## Supporting information

S1 Fig Detailed diagram of copy number profile curves and cAUC. (a) Example of CNV data in standard PLINK format. (b), (d) and (f) Examples of copy number (CN) profile curves illustrating the cAUC between individuals with overlapping duplications. (c) and (e) Examples of CN profile curves illustrating the cAUC between individuals with overlapping deletions. (g) The total cAUC between two individuals with multiple overlapping regions is the sum of multiple areas.
(PDF)

S2 Fig Results of TGP-WG simulation with causal dosage × length effects. (PDF)

S3 Fig Results of TGP-WG simulation with causal dosage effects. (PDF)

S4 Fig Quantile-quantile (QQ) plots for CONCUR p-values from negative control CNV analyses using Taiwan Biobank data. (a) QQ-plot for CONCUR p-values in TWB lipid metabolism pathways negative control analysis. (b) QQ-plot for CONCUR p-values in TWB chromosome 13 negative control analysis.
(PDF)

S1 Table Summary of simulation designs and scenarios. (PDF)

S1 Appendix Proof of symmetry and positive semi-definiteness of cAUC kernel. (PDF)

S2 Appendix Details of TwinGene pseudo CNV data whole genome (TGP-WG) simulation design. (PDF)

S3 Appendix Post-hoc pathway analysis of Taiwan Biobank CNV data in lipid metabolism pathway hsa01040. (PDF)

S4 Appendix Numerical data for figures and tables. (ZIP)
