# Supplementary material for: Association test using Copy Number Profile Curves (CONCUR) enhances power in rare copy number variant analysis
Source: PLoS Comput Biol. 2020 May 4;16(5):e1007797. doi: 10.1371/journal.pcbi.1007797 (PMC7224564; doi:10.1371/journal.pcbi.1007797)
Supplement: S1 Fig — (a) Example of CNV data in standard PLINK format. (b), (d) and (f) Examples of copy number (CN) profile curves illustrating the cAUC between individuals with overlapping duplications. (c) and (e) Examples of CN profile curves illustrating the cAUC between individuals with overlapping deletions. (g) The total cAUC between two individuals with multiple overlapping regions is the sum of multiple areas. (PDF) [file pcbi.1007797.s001.pdf]

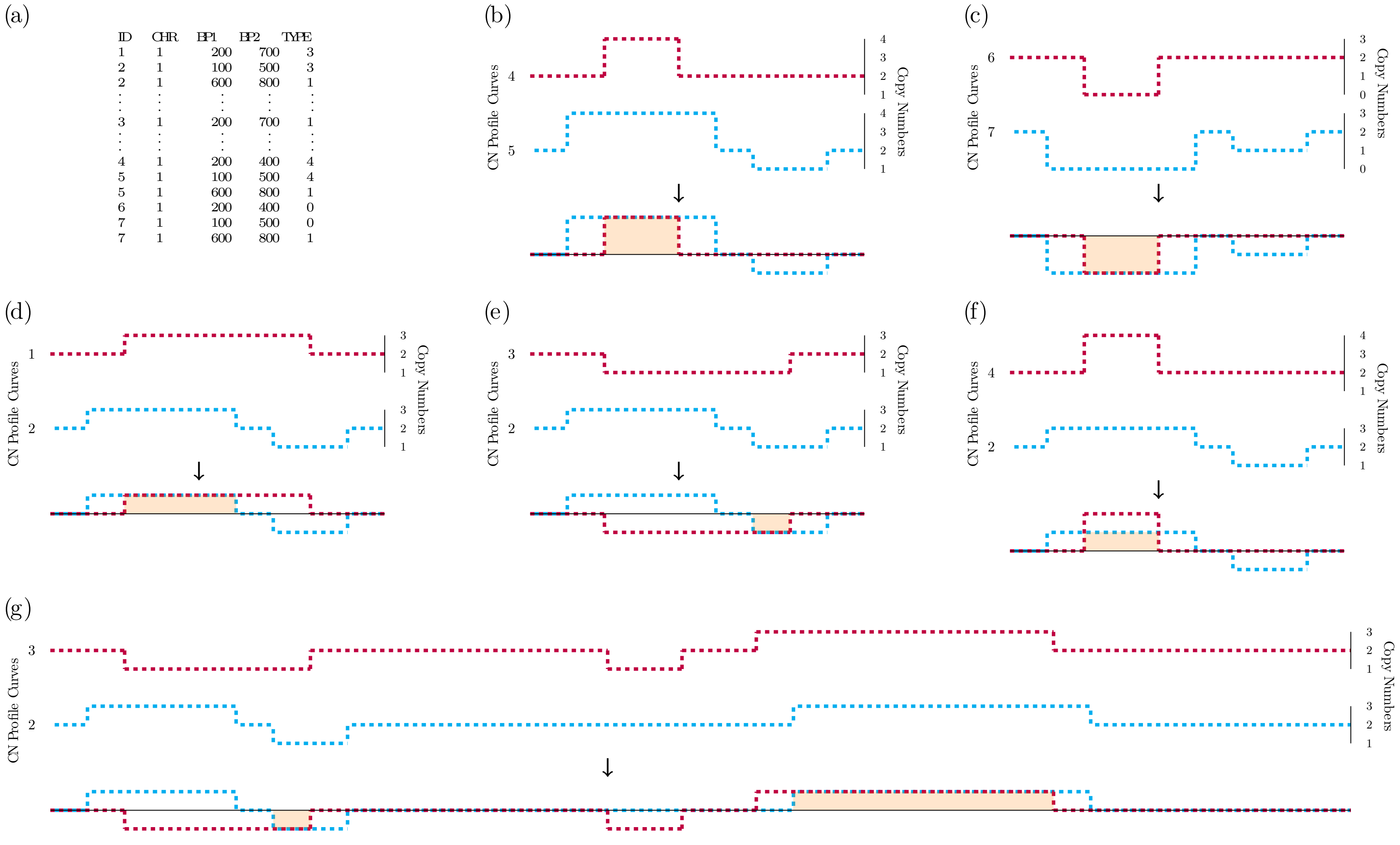

ID	CHR	BP1	BP2	TYPE
1	1	200	700	3
2	1	100	500	3
2	1	600	800	1
⋮	⋮	⋮	⋮	⋮
3	1	200	700	1
⋮	⋮	⋮	⋮	⋮
4	1	200	400	4
5	1	100	500	4
5	1	600	800	1
6	1	200	400	0
7	1	100	500	0
7	1	600	800	1

S1 Fig. Diagram of copy number profile curves and common area under the curve. (a) Example of CNV data in standard PLINK format describing profiles of individuals in a small region of chromosome 1. (b)&(c) Copy number (CN) profile curves illustrating the cAUC between individuals with overlapping duplications of dosage 4 in (b) and individuals with overlapping deletions of dosage 0 in (c). (d)&(e) CN profile curves illustrating the cAUC between individuals with overlapping duplications of dosage 3 in (d) and individuals with overlapping deletions of dosage 1 in (e). (f) CN profile curves illustrating the cAUC between individuals with overlapping duplications of dosage 3 and 4. (g) CN profile curves which contain overlapping CNVs in multiple locations, so that the cAUC between the individuals is the sum of the two areas.
