# Supplementary material for: Association test using Copy Number Profile Curves (CONCUR) enhances power in rare copy number variant analysis
Source: PLoS Comput Biol. 2020 May 4;16(5):e1007797. doi: 10.1371/journal.pcbi.1007797 (PMC7224564; doi:10.1371/journal.pcbi.1007797)
Supplement: S2 Fig — (PDF) [file pcbi.1007797.s002.pdf]

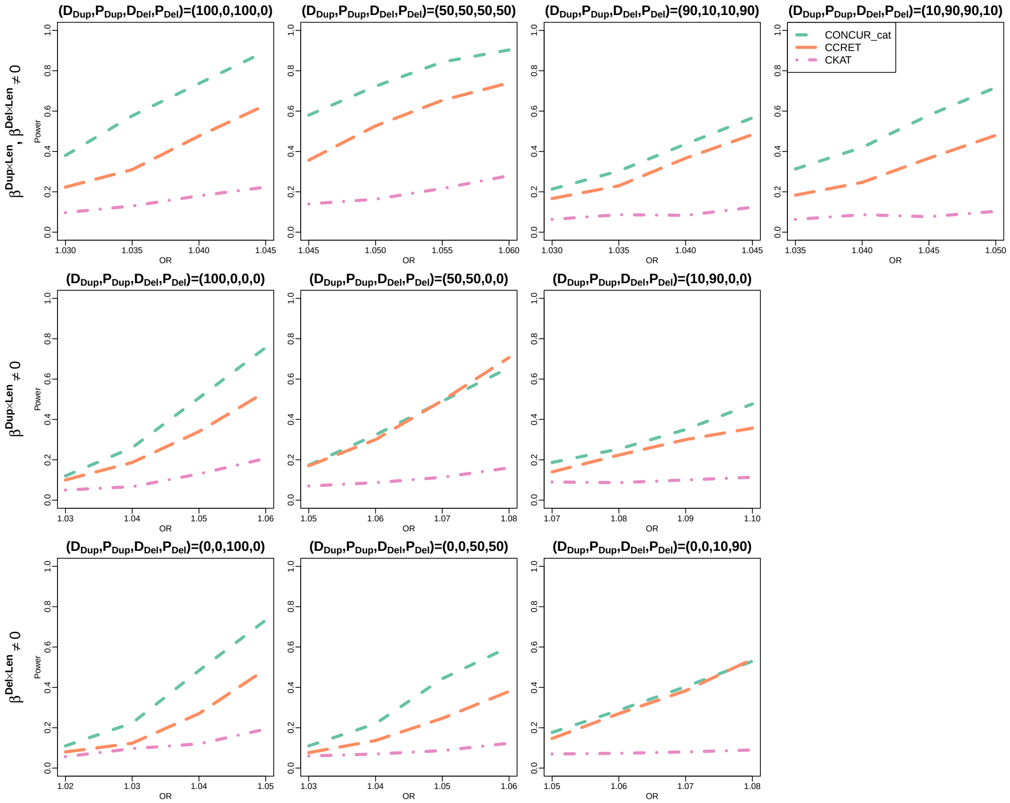

S2 Fig. Results of TGP-WG simulation with causal dosage×length effects. This figure shows the power comparison between CONCUR, CCRET, and CKAT in the TGP-WG simulations (causal dosage×length effects across the genome of TGP data). The top panel shows power under combined duplication and deletion effects, the middle panel shows power under effects from duplications only, and the bottom panel shows power under effects from deletions only. Different proportions of deleterious vs. protective effects are considered as indicated by $(D_{Dup},P_{Dup},D_{Del},P_{Del})$ with D_{Dup} and P_{Dup} reflecting the proportions of deleterious and protective segments among causal duplication segments, and with D_{Del} and P_{Del} defined similarly for causal deletion segments.
