# Supplementary material for: Association test using Copy Number Profile Curves (CONCUR) enhances power in rare copy number variant analysis
Source: PLoS Comput Biol. 2020 May 4;16(5):e1007797. doi: 10.1371/journal.pcbi.1007797 (PMC7224564; doi:10.1371/journal.pcbi.1007797)
Supplement: S4 Fig — (a) QQ-plot for CONCUR p-values in TWB lipid metabolism pathways negative control analysis. (b) QQ-plot for CONCUR p-values in TWB chromosome 13 negative control analysis. (PDF) [file pcbi.1007797.s004.pdf]

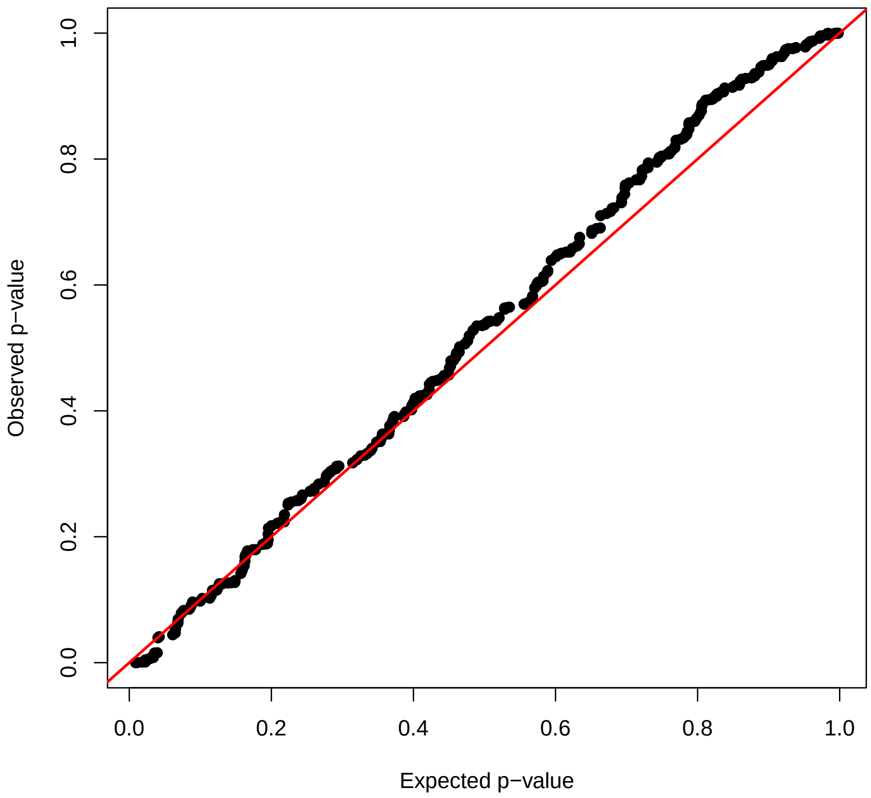

S4 Fig (a). QQ-plot for CONCUR p-values in TWB lipid metabolism pathways negative control analysis. P-values from pathway analyses with permuted TG levels, with 20 replications per pathway (20*15 p-values). Expected p-values come from a Uniform(0,1) distribution.

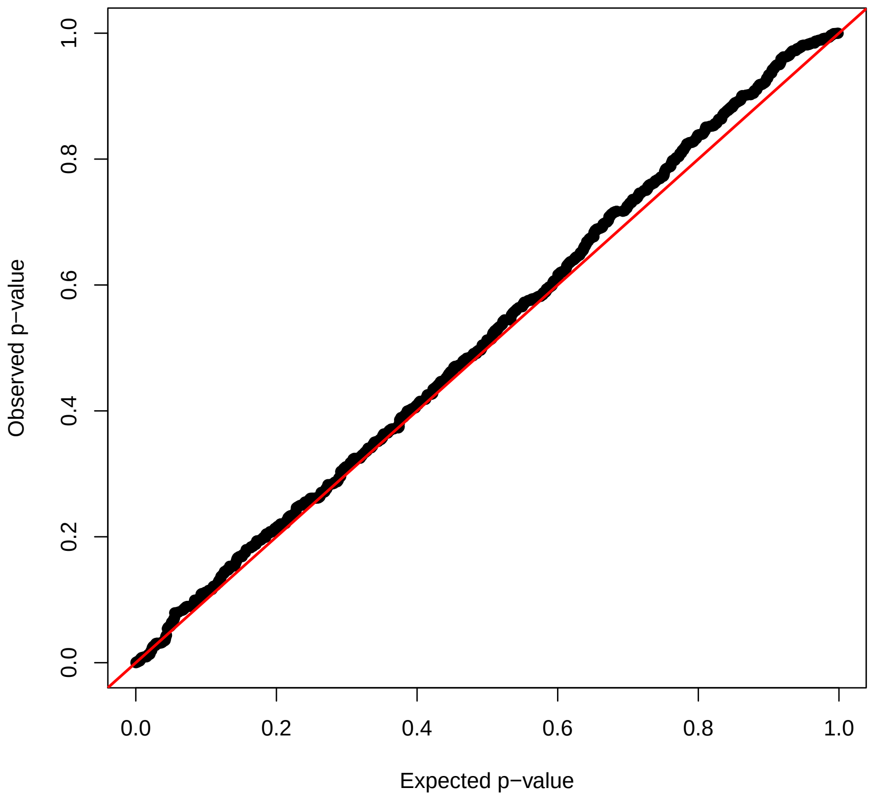

S4 Fig (b). QQ-plot for CONCUR p-values in TWB chromosome 13 negative control analysis. P-values from 1000 replications of the chromosome 13 analysis with permuted TG levels. Expected p-values come from a Uniform(0,1) distribution.
