# Supplementary material for: Association test using Copy Number Profile Curves (CONCUR) enhances power in rare copy number variant analysis
Source: PLoS Comput Biol. 2020 May 4;16(5):e1007797. doi: 10.1371/journal.pcbi.1007797 (PMC7224564; doi:10.1371/journal.pcbi.1007797)
Supplement: S1 Table — (PDF) [file pcbi.1007797.s005.pdf]

Brucker et al. (2020) ***Association Test Using Copy Number Profile Curves (CONCUR) Enhances Power in Rare Copy Number Variant Analysis.***

S1 Table. Summary of Simulation Designs and Scenarios.

Data	Simulation	Results (Tag)	Key features considered in the simulation	Heterogeneity Models (D_{Dup},P_{Dup},D_{Del},P_{Del})
TwinGene pseudo-CNV data	Whole Geneome (TGP-WG)	S2 Fig	• Performance under different causal effect patterns (causal effects from duplication and/or deletion, and different % of deleterious vs. protective effects among causal CNV segments)<br>• Causal effect from length×dosage, where dosage values are categorical<br>• Baseline disease rate = 12%	• Combined Effects: (100,0,100,0); (50,50,50,50); (90,10,10,90); (10,90,90,10)<br>• Duplications: (100,0,0,0); (50,50,0,0); (10,90,0,0)<br>• Deletions: (0,0,100,0); (0,0,50,50); (0,0,10,90)
		S3 Fig	Same as above but with causal effects from dosage alone, where dosage values are categorical	• Combined Effects: (100,0,100,0); (50,50,50,50); (90,10,10,90); (10,90,90,10)<br>• Duplications: (100,0,0,0); (50,50,0,0); (10,90,0,0)<br>• Deletions: (0,0,100,0); (0,0,50,50); (0,0,10,90)
	Chr1 (TGP-Chr1)	Fig 2 (TGP-Chr1(a))	• Performance under different causal effect patterns (causal effects from duplication and/or deletion, and different % of deleterious vs. protective effects among causal CNVs)<br>• Causal effect from length×dosage, where dosage values are categorical<br>• Baseline disease rate = 12%<br>• Compare CONCUR with integer dosage vs. CONCUR with categorical dosage	• Combined Effects: (90,10,90,10); (90,10,10,90); (10,90,90,10)<br>• Duplications: (90,10,90,10); (50,50,0,0); (10,90,0,0)<br>• Deletions: (90,10,90,10); (0,0,50,50); (0,0,10,90)<br>} Basic setting I
		Fig 3a (TGP-Chr1(b))	Same as “basic setting I” but with called CNV boundaries not perfectly aligned with causal region.	• Combined Effects: (90,10,90,10); (90,10,10,90); (10,90,90,10)
		Fig 3b (TGP-Chr1(c))	Same as “basic setting” but with baseline disease rate = 5%	• Combined Effects: (90,10,90,10); (90,10,10,90); (10,90,90,10)
Taiwan Biobank CNV data	Chr1 (TWB-Chr1)	Fig 4 (TWB-Chr1(a))	• Performance under different causal effect patterns (causal effects from duplication and/or deletion, and different % of deleterious vs. protective effects among causal CNVs)<br>• Causal effect from length×dosage, where dosage values are **continuous**<br>• Baseline disease rate = 12%<br>• Compare CONCUR with **continuous dosage** vs. CONCUR with **categorical dosage**	• Combined Effects: (90,10,90,10); (90,10,10,90); (10,90,90,10)<br>• Duplications: (90,10,90,10); (50,50,0,0); (10,90,0,0)<br>• Deletions: (90,10,90,10); (0,0,50,50); (0,0,10,90)<br>} Basic setting II
		Fig 5 (TWB-Chr1(b))	Same as “basic setting II” except using categorical dosage values (instead of continuous dosage values) to simulate phenotypes	• Combined Effects: (90,10,90,10); (90,10,10,90); (10,90,90,10)
