# Supplementary material for: Association test using Copy Number Profile Curves (CONCUR) enhances power in rare copy number variant analysis
Source: PLoS Comput Biol. 2020 May 4;16(5):e1007797. doi: 10.1371/journal.pcbi.1007797 (PMC7224564; doi:10.1371/journal.pcbi.1007797)
Supplement: S1 Appendix — (PDF) [file pcbi.1007797.s006.pdf]

S1 Appendix. Proof of symmetry and positive semi-definiteness of cAUC kernel

Brucker et al. (2020) ***Association Test Using Copy Number Profile Curves (CONCUR) Enhances Power in Rare Copy Number Variant Analysis***

In order to show that $k_{cAUC}(\cdot,\cdot)$ is a valid kernel function, we need to show the associated kernel matrix is symmetric and positive semi-definite (PSD). The cAUC kernel function is given by

$$k_{cAUC}(Z_i, Z_j) = \sum_{k=1}^{22} \int_{\mathbb{N}} \left[\min\left(f_{ik}^{Dup}(x), f_{jk}^{Dup}\right) + \min\left(f_{ik}^{Del}(x), f_{jk}^{Del}(x)\right)\right] \mathrm{d}\mu(x) \tag{1}$$

where Z_i and Z_j represent the input data matrices containing CNV chromosome, starting and end locations, and dosage information for all CNVs in the profiles of individuals i and j, for $i, j = 1, \cdots, n$ individuals; where $f_{ik}^{Dup}(x)$ and $f_{ik}^{Del}(x)$ are the duplication profile curve and deletion profile curve of individual i on chromosome k; and $\mu(x)$ is the counting measure. The derivation of the duplication and deletion profile curves as a function of the input data Z_i is detailed in the manuscript Methods section. We define the $n \times n$ kernel matrix $\mathbf{K}^{Dup}$ such that its (i, j)th entry is given by

$$\mathbf{K}_{ij}^{Dup} = \sum_{k=1}^{22} \int_{\mathbb{N}} \min\left(f_{ik}^{Dup}(x), f_{jk}^{Dup}(x)\right) \mathrm{d}\mu(x) \tag{2}$$

for $i, j = 1, \cdots, n$, with $\mathbf{K}^{Del}$ defined similarly so that $\mathbf{K} = \mathbf{K}^{Dup} + \mathbf{K}^{Del}$.

It is sufficient to show that $\mathbf{K}^{Dup}$ is symmetric and PSD. Define f_i to be $f_{ik}^{Dup}(x)$ at a fixed value of x on a fixed chromosome k and likewise for f_j. Then define the function

$$k(f_i, f_j) = \min(f_i, f_j) \tag{3}$$

and a corresponding kernel matrix K with its (i, j)th element $K_{ij} = k(f_i, f_j)$. Note that $\mathbf{K}_{ij}^{Dup}$ is simply the sum of K_{ij} across all x in a chromosome across all chromosomes $k = 1, \cdots, 22$. Therefore, if K is symmetric and PSD, so is the $\mathbf{K}^{Dup}$.

First, it is evident that K is symmetric kernel, since the minimum operator is symmetric. Now, to show that K is PSD, we must show that

$$\sum_{i=1}^{N} \sum_{j=1}^{N} \alpha_i \alpha_j k(f_i, f_j) \geq 0 \tag{4}$$

$\forall \boldsymbol{\alpha} = (\alpha_1, \cdots, \alpha_n)$ and for all possible $f_i, f_j \geq 0$. Call the domain of the duplication profile curves

$\mathbb{F}$. Then we have

$$\sum_{i=1}^{N}\sum_{j=1}^{N}\alpha_i\alpha_j \min(f_i, f_j) = \sum_{i=1}^{N}\sum_{j=1}^{N}\int_{\mathbb{F}}\alpha_i\alpha_j \ \mathbb{1}_{t\le f_i}(t)\mathbb{1}_{t\le f_j}(t) \ \mathrm{d}t \tag{5}$$

$$= \int_{\mathbb{F}}\left(\sum_{i=1}^{N}\alpha_i\mathbb{1}_{t\le f_i}(t)\right)\left(\sum_{j=1}^{N}\alpha_j\mathbb{1}_{t\le f_j}(t)\right)\mathrm{d}t \tag{6}$$

$$= \int_{\mathbb{F}} G(t)^2\mathrm{d}t \ge 0 \tag{7}$$

where $\mathbb{1}_{t\le f_i}(t)$ is a function that takes value 1 if $t \le f_i$ and value 0 otherwise, and $G(t) = \sum_{i=1}^{N}\alpha_i\mathbb{1}_{t\le f_i}(t)$. Hence K is positive semi-definite and therefore so is $\mathbf{K}^{Dup}$, and $\mathbf{K}^{Del}$ by the same logic.

Further notes on the kernel matrix K

In the case of a study that includes individuals with no CNV events across the entire genome, following the above definition of the cAUC kernel function, these individuals would have a self-similarity of 0, i.e., $k_{cAUC}(Z_i, Z_i) == 0$, and results in some 0 values in the diagonal of the kernel matrix $\mathbf{K}$ (i.e., diagonal among these individuals). To fix this issue, we overwrite 0 diagonal values to be the minimum of all non-zero diagonal values in $\mathbf{K}$, i.e., the minimum self-similarity among all individuals with CNV events. In both the simulations and real data analysis, we found that kernels with zeros on the diagonal and kernels with zeros overwritten as above produced identical p-values to 10 decimal places. We implement the zero-overwriting approach in the CONCUR software though it has little impact on the analysis results.
