# Supplementary material for: Association test using Copy Number Profile Curves (CONCUR) enhances power in rare copy number variant analysis
Source: PLoS Comput Biol. 2020 May 4;16(5):e1007797. doi: 10.1371/journal.pcbi.1007797 (PMC7224564; doi:10.1371/journal.pcbi.1007797)
Supplement: S2 Appendix — (PDF) [file pcbi.1007797.s007.pdf]

S2 Appendix. Details of TwinGene pseudo CNV data whole genome (TGP-WG) simulation design

Brucker et al. (2020) ***Association Test Using Copy Number Profile Curves (CONCUR) Enhances Power in Rare Copy Number Variant Analysis***

In the TGP-WG simulations, we examined the methods' performance in two main scenarios: under a dosage×length signal and under a dosage-only signal. Within each scenario, we considered three sub-scenarios with different causal effects: causal effects from both duplications and deletions, causal effects from duplications only, and causal effects from deletions only. In each sub-scenario, we designated varying percentages of the causal segments to be deleterious (D) or protective (P). When both duplication and deletion were causal, the settings included $(\mathrm{D}_{\mathrm{Dup}}, \mathrm{P}_{\mathrm{Dup}}, \mathrm{D}_{\mathrm{Del}}, \mathrm{P}_{\mathrm{Del}}) = (100, 0, 100, 0)$, $(50, 50, 50, 50)$, $(90, 10, 10, 90)$ and $(10, 90, 90, 10)$. In scenarios where duplications (or deletions) alone are causal, possible settings included $(\mathrm{D}_{\bullet}, \mathrm{P}_{\bullet})$=(100,0), (50,50), and (10,90).

Among the CNV segments across the genome, we selected 200 segments to be causal, which consist of 100 causal "dup-segments" with at least one duplication and another 100 causal "del-segments" with at least one deletion. A causal dup-segment cannot be a causal del-segment. These causal segments were chosen as a random draw of 50 pairs of adjacent segments which both contained duplications, and another 50 pairs of adjacent segments which both contained deletions. This adjacent causal segment approach was designed to ensure that causal regions had more realistic lengths, since some segments were very short by chance.

The implementation of the CONCUR_cat, CCRET, and CKAT methods is given in the main text. The power was evaluated in the range of odds ratios ($\exp(\beta)$) 1.02-1.10 in the scenarios with dosage×length effects and 1.1-1.9 in the scenarios with dosage-only effects. Power estimates are reported for a range of effect sizes such that the power ranges roughly from 0.2 to 0.8.
