# Supplementary material for: Association test using Copy Number Profile Curves (CONCUR) enhances power in rare copy number variant analysis
Source: PLoS Comput Biol. 2020 May 4;16(5):e1007797. doi: 10.1371/journal.pcbi.1007797 (PMC7224564; doi:10.1371/journal.pcbi.1007797)
Supplement: S3 Appendix — (PDF) [file pcbi.1007797.s008.pdf]

S3 Appendix. Post-hoc pathway analysis of Taiwan Biobank CNV data in lipid metabolism pathway hsa01040

Brucker et al. (2020) ***Association Test Using Copy Number Profile Curves (CONCUR) Enhances Power in Rare Copy Number Variant Analysis***

To illustrate possible CONCUR post hoc analyses to probe the potential sources of the pathway-level signals found in the TWB analysis, we looked more closely at one pathway, hsa01040 (biosynthesis of unsaturated fatty acids), for which both CONCUR and CKAT were significant while CCRET was borderline significant. Previous studies have reported that monounsaturated fat acids or polyunsaturated fatty acids can effect TG levels [1, 2]. Given the major function of the genes in hsa01040 (i.e., the biosynthesis of unsaturated fatty acids), it is not unexpected that CNVs in these genes were significantly associated with TG levels. In Table A, we reported summary statistics describing CNV length and dosage in hsa01040 for individuals with different levels of TG. Based on the TG quantiles from the sample data, we classified individuals as having high TG (>75th percentile [>140 mg/dL]), medium TG (25th−75th percentile [68-140 mg/dL]) and low TG (<25th percentile [<68 mg/dL]). We applied ANOVA to detect differences in CNV length and in dosage characteristics, and applied chi-squared tests to assess differences in the proportion of individuals with CNVs across TG levels. In addition, we examined CNV features in all CNVs together and in duplications and deletions separately.

Taking p-values < 0.05 as suggestive of a “promising” association with TG, we did not observe any CNV associations when all CNVs were analyzed together; but for duplications only, there were promising differences in CNV length (p-value=0.0063) and weaker differences in dosage (p-value=0.0255) across TG levels. There were also some weak significance in CNV length for deletions (p-value=0.0423). We were cautious to not over-interpret these “promising” associations since this stratified analysis reflected only marginal associations of a

Table A. Descriptive statistics for hsa01040 pathway. TG values are classified as Low (<the 25th percentile [<68 mg/dL]; n=2,931), Medium (the middle 50% [68 - 140 mg/dL]; n=5,844), and High (>the 75th percentile [>140 mg/dL]; n=2,889). The percent of individuals with CNVs is with respect to the total number of individuals in each TG category. The mean number of CNVs per individual and mean total length of CNVs (bp) per individual are reported, as well as the mean lengths (bp) and mean dosage per CNV. "Promising" associations with TG are marked with $\star\star$ to indicate p-value< 0.01 and with $\star$ to indicate p-value< 0.05.

CNV Type	TG Level	Pct Individuals with CNV	Mean # CNVs per Individual	# Genes Interrupted	Mean Total CNV Length per Individual (bp)	Mean CNV Length (bp)	Mean CNV Dosage
	Low	6.18%	3.33	23	25143.71	2433.58	1.63
All	Medium	6.07%	3.52	23	24447.30	2473.48	1.63
	High	7.17%	3.48	23	31091.65	2471.43	1.64
	Low	2.8%	5.84	16	29630.62	2590.55*	1.41
Deletion	Medium	2.74%	6.24	17	28107.36	2593.05*	1.40
	High	3.32%	5.79	16	32039.63	2067.96*	1.39
	Low	3.62%	1.17	20	7811.20	1827.24**	2.50*
Duplication	Medium	3.54%	1.22	23	10009.61	2001.81**	2.52*
	High	4.15%	1.38	22	27897.23	3831.02**	2.49*

CNV feature, and the tests did not account for the effect heterogeneity that motivates the application of kernel-based methods. We also applied CONCUR to duplications and deletions separately, and found a very significant association with TG in duplications (p-value $< 1 \times 10^{-8}$) and a weaker signal in deletions (p-value=0.0313).

To further explore the signal from duplications, we visualized CNVs in the 23 genes in hsa01040. Figure A displays duplications and deletions in the CNV profiles of individuals categorized by their TG level (low, medium, and high), with profiles clustered using the Weighted Pair Group Method with Arithmetic Mean (WPGMA) hierarchical clustering method.

For further exploration, we applied CONCUR to duplications in each gene and found that several genes had strong association p-values (i.e., $< 10^{-4}$), *BAAT, ELOVL4, ELOVL6, ELOVL5, HSD17B4*, and *SCD5*. Notably, *BAAT* is an amino acid N-acyltransferase

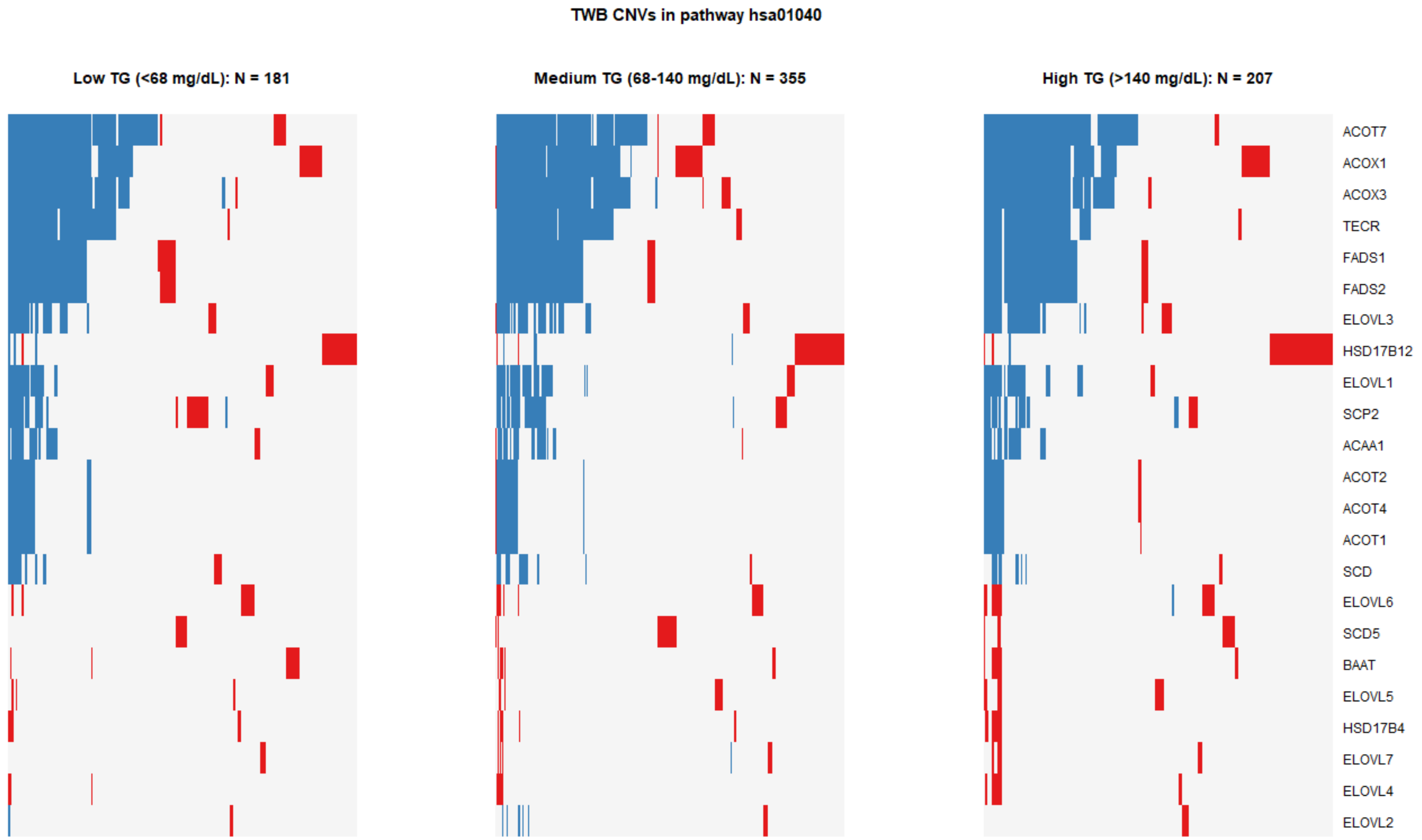

Figure A. Visualization of CNV activity in pathway hsa01040 by level of triglycerides (TG). CNV activity in genes in hsa01040 is shown by level of TG (Low, Medium, and High), with duplications in red and deletions in darkolive. Columns represent individuals, and genes shown here are the 23 genes in the pathway that contain CNVs, ordered by the number of CNVs contained therein.

for bile acid. Previous studies have demonstrated that bile acids are important regulators for TG level through crosstalk with farnesoid X receptor (FXR) [3, 4]. Since conversion of cholesterol to bile acid is an essential step in preventing the accumulation of TG, copy number duplications in *BAAT* may directly affect TG levels in the blood. Three *ELO* genes had significant CNV associations. Since the major functions of these genes focus on the elongation of fatty acids, CNV events in these genes are likely to affect the production and metabolism of TG. For example, one study showed that hepatic steatosis was observed in *ELOVL5*-knockout mice due to the activation of SREBP-1c and its target genes [9]. *HSD17B4* is a dehydrogenase, which is able to inhibit the production of DHEA[5]. A previous study showed that TG levels were inversely correlated to DHEA levels in men with type 2 diabetes [6], suggesting a potential link between CNVs in *HSD17B4* and TG levels. *SCD5* serves as a critical enzyme providing a double bond to construct complex lipid molecules such as TG [7, 8], and thus dysregulation of *SCD5* expression may impact TG levels.

Further analyses are required to formally localize the sources of the CNV association signal in this pathway and others, but this exploratory analysis nonetheless serves to enrich our understanding of the association in pathway hsa01040 through examination of CNV-level and gene-level features.

References

[1] Grundy SM. Monounsaturated fatty acids and cholesterol metabolism: implications for dietary recommendations. J Nutr. 1989;119(4):529-533.

[2] Ooi EM, Watts GF, Ng TW, et al. Effect of dietary Fatty acids on human lipoprotein metabolism: a comprehensive update. Nutrients. 2015;7(6):4416-25.

[3] Lien F, Berthier A, Bouchaert E, et al. Metformin interferes wtih bile acid homeostasis through AMPK-FXR crosstalk. J Clin Invest. 2014;124(3):1037-1051.

[4] Watanabe M, Houten SM, Wang L, et al. Bile acids lower triglyceride levels via a pathway involving FWR, SHP, and SREBP-1c. J Clin Invest. 2004;112(10):1408-18.

[5] de Launoit Y, Adamski J. Unique multifunctional HSD17B4 gene product: 17beta-hydroxysteroid dehydrogenase 4 and D-3-hydroxyacyl-coenzyme A dehydrogenase/hydratase involved in Zellweger syndrome. J Mol Endocrinol. 1999;22(3):227-40.

[6] Boudou P, de Kerviler E, Erlich D, et al. Exercise training-induced triglyceride lowering negatively correlates with DHEA levels in men with type 2 diabetes. Int J Obes Relat Metab Disord. 2001;25(8):1108-12.

[7] Castro LF, Wilson JM, Gonçalves O, et al. The evolutionary history of the stearoyl-CoA desaturase gene family in vertebrates. BMC Evol Biol. 2001;11:132.

[8] Flowers MT, Ntambi JM. Role of stearoyl-coenzyme A desaturase in regulating lipid metabolism. Curr Opin Lipidol. 2008;19(3):248-256.

[9] Sassa T, Kihara A. Metabolism of very long-chain Fatty acids: genes and pathophysiology. Biomol Ther (Seoul). 2014;22(2):83-92.
